# Green fluorescent protein nanopolygons as monodisperse supramolecular assemblies of functional proteins with defined valency

**DOI:** 10.1038/ncomms8134

**Published:** 2015-05-14

**Authors:** Young Eun Kim, Yu-na Kim, Jung A. Kim, Ho Min Kim, Yongwon Jung

**Affiliations:** 1 Department of Chemistry, KAIST, 291 Daehak-ro, Yuseong-gu, Daejeon 305-701, Korea; 2 Graduate School of Nanoscience and Technology, KAIST, Daejeon 305-701 Korea; 3 Graduate School of Medical Science and Engineering, KAIST, Daejeon 305-701, Korea

## Abstract

Supramolecular protein assemblies offer novel nanoscale architectures with molecular precision and unparalleled functional diversity. A key challenge, however, is to create precise nano-assemblies of functional proteins with both defined structures and a controlled number of protein-building blocks. Here we report a series of supramolecular green fluorescent protein oligomers that are assembled in precise polygonal geometries and prepared in a monodisperse population. Green fluorescent protein is engineered to be self-assembled in cells into oligomeric assemblies that are natively separated in a single-protein resolution by surface charge manipulation, affording monodisperse protein (nano)polygons from dimer to decamer. Several functional proteins are multivalently displayed on the oligomers with controlled orientations. Spatial arrangements of protein oligomers and displayed functional proteins are directly visualized by a transmission electron microscope. By employing our functional protein assemblies, we provide experimental insight into multivalent protein–protein interactions and tools to manipulate receptor clustering on live cell surfaces.

## Abstract (web summary)

Supramolecular protein assemblies can provide novel nano-architectures with diverse structures and functions. Here, the authors report a fabrication strategy for a series of monodisperse protein oligomers, which allows valency-controlled display of various functional proteins.

## Graphical abstract

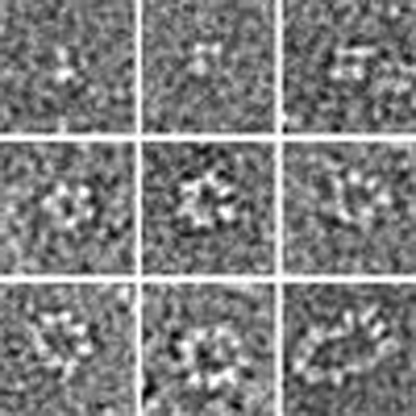

Biomolecules are extremely attractive building blocks for designing novel nano-architectures with molecular precision. DNA self-assembly through specific basepairing, known as DNA origami, has in particular allowed the creation of an array of programmed structures^1^,^2^. In contrast, precise design of protein assemblies, such as natural protein fibres or layers^3^,^4^, has been highly challenging due to the structural complexities of proteins, despite their highly diverse functionalities^5^,^6^,^7^. The spatial organization of functional proteins in various but well-defined nanostructures (for example, in cubes^8^ or lattices^9^) is a key objective in protein nanotechnology. Although several studies have reported symmetric assemblies of multimeric protein subunits with atomic level accuracy by computational design^8^,^10^,^11^,^12^,^13^,^14^, available structures are still limited and functionalization has not been implemented.

To fully understand and use the collective (multivalent) properties of assembled proteins, the number of protein-building blocks must also be precisely controlled. In this sense, protein assemblies need to be prepared in a monodisperse population, providing discrete protein polymers with a systemically varied number of protein monomers. Several sophisticated assembling strategies have been reported for the construction of artificial supramolecular protein polymers, which form various structures ranging from protein wires and rings to even lattices^9^,^15^,^16^,^17^,^18^. These protein polymers were assembled by diverse types of molecular recognitions, including metal ion–protein, enzyme–inhibitor, protein–peptide and protein–cofactor interactions^9^,^16^,^17^,^18^,^19^, or by chemical/enzymatic linkages^20^,^21^,^22^,^23^. However, most current strategies produce protein assemblies with polydisperse distributions in their oligomeric states. Fabrication of homogeneously populated protein oligomers will be an essential step for in-depth understanding and applications of supramolecular protein assemblies, and this monodispersity will also provide greater opportunities to obtain accurate structural information on novel protein nano-assemblies, as demonstrated with computationally designed protein assemblies reported in recent studies^8^,^10^,^14^,^24^.

Here we report the first example of a set of discrete (monodisperse) protein oligomers with well-defined polygonal structures, which allows spatially accurate and valency-controlled display of various functional proteins. Green fluorescent protein (GFP) is engineered to be self-assembled as translated inside cells, producing a mixture of GFP oligomers with various sizes. Protein precipitation (a major problem of large artificial protein assemblies) of these GFP oligomers is prevented by systematic introduction of negative charges on the GFP surface. More importantly, these supercharges on GFP enable DNA-like gel-based purification of monodisperse GFP oligomers, the polygonal arrangement of which is directly visualized by transmission electron microscopy (TEM). In addition to these GFP (nano)polygons, linearly opened GFP oligomers are also constructed by modifying the cellular GFP assembly. Several functional proteins are successfully displayed on GFP polygons by simple genetic fusion. Proteins are precisely positioned in these polygonal spaces with defined orientation and number of protein units. Finally, we employ (antibody binding) protein G-functionalized polygons to investigate multivalent protein–antibody interactions and to control the level of antibody-mediated receptor clustering on the cell surface.

## Results

### Cellular self-assembly of GFP oligomers

To construct functionally versatile and structurally defined protein supramolecular structures, we exploited the previously developed split ‘superfolder' GFP system^25^,^26^. The β-strand 11 of GFP (GFP 11, amino acids 215–230) spontaneously assembles with truncated GFP 1–10 (GFP 1–10, amino acids 1–214) to form a fluorescently matured GFP. These split fragments feature a unique peptide–protein interaction^27^, which can be highly beneficial for protein self-assembly. As the interaction is non-covalent but nearly irreversible, resulting protein assemblies may withstand multiple environmental changes during purification/separation, characterization and even potential multistep applications. In addition, this fully protein-based interaction will allow protein assembly in cells, circumventing the need for coupling ligands or chemical/enzymatic reactions and permitting diverse functionalization via genetic fusion. GFP itself is also an attractive building block with a wide range of available structural and functional variants^28^,^29^ and the intrinsic fluorescence signal.

**Figure 1:**
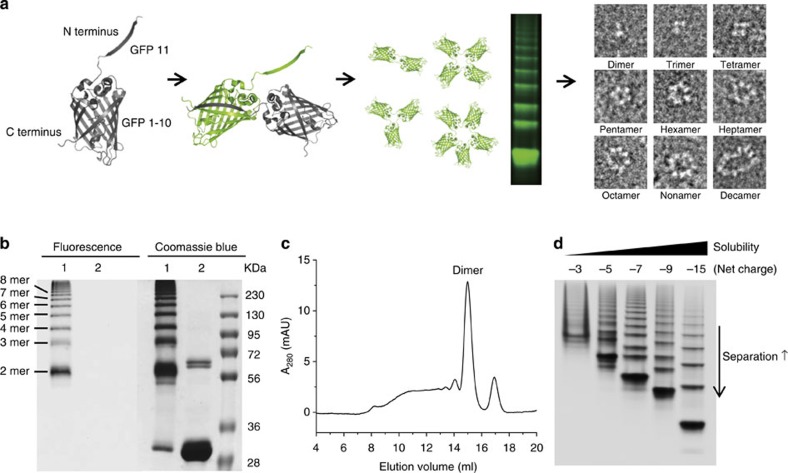
Cellular self-assembly of GFP oligomers. (**a**) Schematic representation of the fabrication of discrete GFP (nano)polygons. The β strand 11 of GFP is connected to the N terminus of GFP 1–10 using a short peptide linker, and the resulting GFP monomer unit undergoes self-assembly into polygonal structures in the cell. Through the introduction of supercharges on the polygon surface, GFP polygon mixtures can be separated and isolated depending on the number of GFP-building blocks, to produce a series of discrete GFP polygons. (**b**) SDS–PAGE analysis of GFP oligomers. Oligomer mixtures were applied to a PAGE gel containing 0.1% SDS without (lane 1) or with (lane 2) boiling. The gel was analysed by a fluorescent image analyser with 470-nm excitation and 530-nm emission filters (left), and Coomassie blue staining (right). (**c**) SEC of GFP oligomers using a Superdex 200 column (10/300 GL). (**d**) Native PAGE analysis of GFP oligomer charge variants with net charges of −3, −5, −7, −9 and −15. Enhanced solubility and gel separation of the oligomers are indicated.

The strategy for cellular self-assembly of GFP oligomers is schematically represented in Fig. 1a. We designed an assembling GFP monomer to be the smallest possible unit for higher cellular production and further incorporation of functional proteins. The GFP 11 peptide was genetically linked to the amino-terminal end of GFP 1–10 via a small peptide linker. The peptide linker was carefully designed to avoid intramolecular association between GFP 11 and GFP 1–10 in the same GFP monomer, granting only intermolecular self-assemblies. The designed GFP monomer was well-expressed in cells and successfully underwent cellular self-assembly to form GFP oligomers with a size range from dimer to over decamer (Fig. 1). Different peptide linkers between GFP 1–10 and GFP 11 were investigated with varying length, rigidity, charge and size (Supplementary Table 1 and Supplementary Fig. 1). The length of the linker was the most critical factor for cellular self-assembly. A flexible tri-peptide (GGT) linker provided maximal protein expression and polymerization, while avoiding intramolecular GFP formation. Resulting GFP oligomers were produced in an over 100-mg scale from 1 litre of *E. coli* flask culture and easily purified using Ni-affinity chromatography.

Size distributions of the assembled GFP oligomers were analysed by polyacrylamide gel electrophoresis (PAGE; Fig. 1b) and size-exclusion chromatography (SEC; Fig. 1c). GFP oligomer mixtures were not clearly separated by a native PAGE analysis, probably due to low surface charges of GFP (net charge −3). High stability of the present GFP assemblies, however, allowed a PAGE analysis with added denaturing detergent, SDS, for size-dependent separation, although longer incubation with SDS eventually destabilized protein oligomers. Multiple bands of large-sized GFP oligomers were observed by both fluorescence imaging and Coomassie blue staining, whereas only GFP monomer was detected after boiling denaturation (Fig. 1b). Although the dimeric form was the main product, a wide range of multimerized GFP oligomers (between 56 and 230 kDa, corresponding to dimers to octamers) were distinctly visualized. GFP oligomers with sizes >230 kDa (>octamer) were also observed. Based on PAGE and SEC data, close to 50% of expressed GFP monomers were polymerized in cells to high-molecular-weight oligomers (>56 kDa, dimer).

One of the major difficulties encountered in the construction of artificial protein polymers is the tendency of large protein assemblies to form irreversible aggregates (and eventually to precipitates), in particular when monomeric protein is forced to assemble naturally. Here, purified GFP oligomers were stable only in the presence of a high concentration of salts (over 500 mM NaCl was required to solubilize 1 mg ml^−1^ of GFP oligomers). To improve the oligomer solubility, we engineered the surface charge of the assembling GFP unit. Previously, various supercharged GFPs carrying a net charge of −30, −25, +36 or +48 were reported with significantly increased resistance against protein precipitation^30^. Surface-exposed amino acids of the GFP monomer were systematically replaced with negatively charged amino acids (Asp or Glu; Supplementary Fig. 2 and Supplementary Table 2). Mutated amino acids were selected from previously engineered residues in super-negative charged GFPs^30^, while excluding essential residues for protein self-assembly^26^. Charge variants of the assembling GFP with different net charges (−5, −7, −9 or −15) were successfully oligomerized to form negatively charged GFP oligomers, although the degree of polymerization was slightly lowered by increased surface charge (Fig. 1d and Supplementary Fig. 3). Importantly, no significant precipitation was observed from supercharged (−7, −9 and −15) GFP oligomers with over 5 mg ml^−1^ protein concentration in PBS buffer (125 mM NaCl).

### Discrete preparation of monodisperse GFP polygons

**Figure 2:**
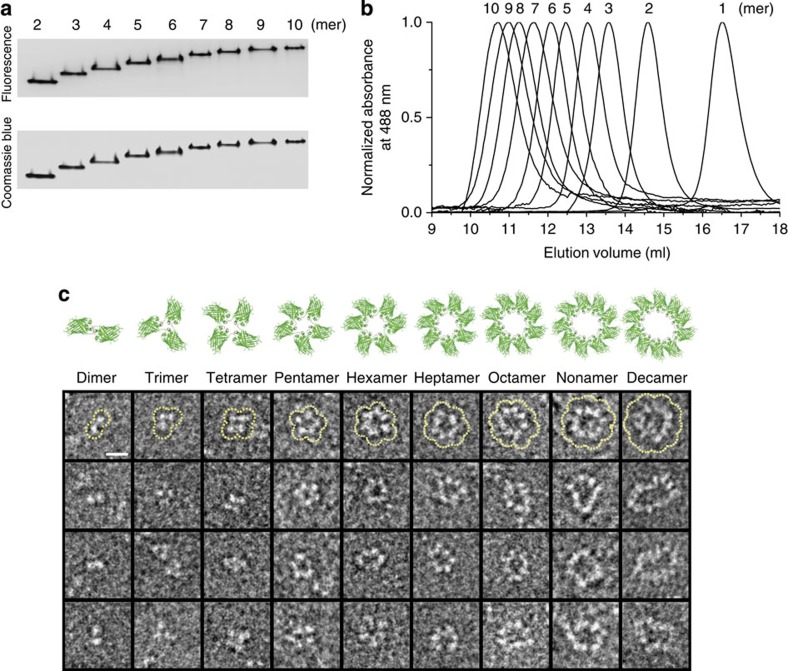
Size-distribution analysis and visualization of discrete GFP polygons. (**a**) Native PAGE analysis of purified GFP polygons from dimer to decamer. The gel was analysed by a fluorescent image analyser (top) and Coomassie blue staining (bottom). (**b**) SEC analysis of discrete GFP polygons from monomer to decamer. Absorbance spectra (at 488 nm) were normalized on their maximum peaks. (**c**) Representative TEM images and schematic drawings of discrete GFP polygons. Polygonal GFP arrangements in TEM images (the first row) were outlined by dotted yellow lines. Scale bar, 10 nm.

An increase of negative charges on GFP oligomers also allowed well-separated and discrete migration of the oligomers in a native PAGE gel (without SDS), depending on the oligomer size (Fig. 1d). Observed protein oligomer separation with a single protein-unit resolution (from dimer to over decamer) in the native condition has not been possible with common chromatographic methods (Fig. 1c and Supplementary Figs 3 and 4). Evenly distributed negative charges on the GFP oligomer surface, similar to those on DNA, probably attribute to this size-dependent separation. We thereby subsequently attempted purification of discrete GFP multimers from oligomer mixtures using a gel electro-elution process, which is also one of major DNA purification methods. Considering polymerization, solubility and gel separation of the oligomers, GFP oligomers with a net charge of −9 were employed for purification. After electrophoresis, fluorescent oligomer bands were excised under ultraviolet light without any staining processes. Fluorescent protein building blocks enabled simple visualization of protein assemblies in native gels, minimizing potential damages to structures and functions of the assemblies during protein labelling. Highly charged oligomers were also well-eluted from the gel on an electric field, and eluted oligomers could be concentrated (over 0.5 mg ml^−1^) and stably stored in PBS. Purified GFP oligomers were reanalysed by native PAGE and SEC (Fig. 2a,b). Highly pure (monodisperse) oligomers from dimer to decamer were obtained with retained multimeric structures as well as fluorescent properties. Approximately 60% of applied protein oligomer mixtures were purified as monodisperse oligomers after gel-based separation and electro-elution. For relatively small oligomers (trimer, tetramer and pentamer), 4–6 mg of each oligomer could be prepared from 1 litre of *E. coli* culture, whereas GFP oligomers from hexamer to decamer could be homogeneously isolated in a 2-to 3-mg scale from the same protein mixtures.

Next, the structures of discrete GFP oligomers were directly investigated by TEM. Each monomeric unit of the GFP oligomers was clearly visualized in TEM images. GFP oligomers from dimers to decamers were rigidly aligned in polygonal nano-structures with widths in a range of 8–20 nm (Fig. 2c and Supplementary Figs 5 and 6). Polygonal structures indicate that the lastly assembled GFP 1–10 is closed by the first GFP 11 peptide (Fig. 1a), to avoid carrying an unstable GFP 1–10 fragment^26^,^27^. Indeed, all GFP monomers in polygons were fluorescently active (Supplementary Fig. 7). Exact numbers of GFP units and strictly polygonal structures were observed for 2-, 3-, 4- and 5-meric GFP oligomers (Supplementary Figs 8 and 9). For larger GFP polygons, a few extended forms of GFP oligomers were also observed (6 mer, 2.8%; 7 mer, 4.6%; 8 mer, 4.5%; 9 mer, 5.8%; 10 mer, 9.5%; Supplementary Figs 10, 11 and 12). Taking these results together, we report a collection of highly ordered and discrete protein nano-assemblies with precisely controlled oligomeric states.

### Multivalent display of functional proteins on GFP polygons

**Figure 3:**
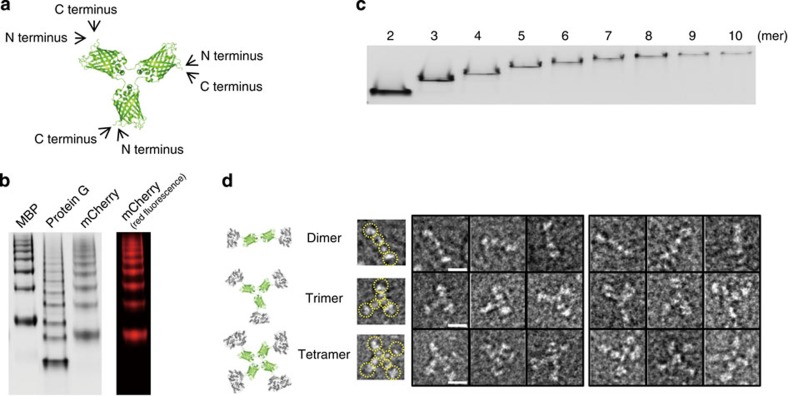
Multivalent display of functional proteins on GFP polygons. (**a**) Schematic drawing of the triangular GFP oligomer with locations of N and C termini. (**b**) Native PAGE analysis of MBP-, protein G- and mCherry-displayed polygons. The gel was analysed by a fluorescent image analyser with 470-nm excitation and 530-nm emission filters (left), and 625-nm excitation and 695-nm emission filters (right). (**c**) Native PAGE analysis of discrete protein G polygons from dimer to decamer. (**d**) Schematic drawing and TEM images of N- (left set of panels) and C- (right set of panels) terminal-fused MBP polygons from dimer to tetramer. MBP and GFP are shown respectively in grey and green. Protein arrangements in TEM images (copies of those in the first column) were indicated with dotted yellow circles. Scale bars, 10 nm.

Developed GFP polygons offer a powerful backbone scaffold for valency-controlled organization of functional proteins with nanoscale accuracy. Functionalization was achieved by simple genetic fusion on the monomeric GFP unit. Both N- and C-terminal fusion sites of the monomer are located on the vertices of the polygons, well separated from the backbone GFPs (Fig. 3a). We introduced various functional proteins including small molecule-binding MBP (maltose-binding protein, 44 kDa), fluorescent mCherry (red fluorescent protein, 25 kDa) and protein-binding protein G (antibody binding protein, 7 kDa). All proteins were successfully displayed on the GFP polygons without interrupting polymerization (Fig. 3b). Only MBP-displayed polygons seemed to have a slightly low degree of polymerization, potentially due to the large size of MBP. All displayed proteins also retained their binding and fluorescence functions (Fig. 3b and Supplementary Fig. 13).

Functionalized polygons could also be purified with monodisperse populations, as illustrated by valency-dependent purification of protein G- (Fig. 3c) and MBP- (Supplementary Fig. 13) displayed polygons. Spatial organization of displayed proteins on backbone GFPs was examined by a TEM analysis. The protein assemblies formed by fusion of GFP polygons to relatively large MBP, which also has a different shape from GFP, were subjected for a structural analysis. TEM images of GFP polygons fused with MBP at both the N and C terminus clearly revealed precise polygonal displays of MBP proteins (Fig. 3d).

### Construction of linearly opened GFP oligomers

**Figure 4:**
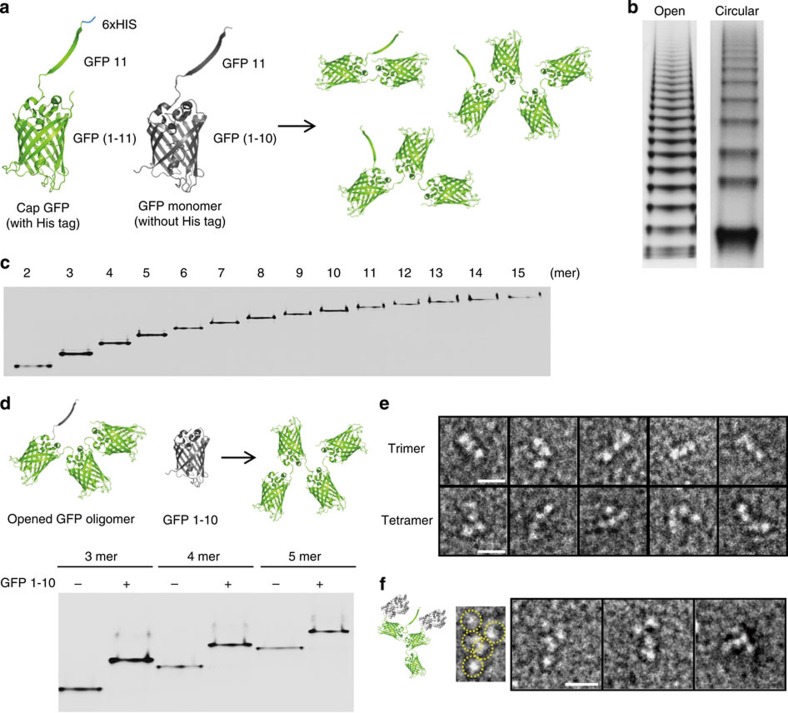
Fabrication and characterization of linearly opened GFP oligomers. (**a**) Schematic representation of the construction of linearly opened GFP oligomers. CapGFP is designed to contain the GFP 11 strand connected to the N terminus of full-length GFP (1–11) with a His tag. Both CapGFP and GFP monomer (without His tag) were co-expressed in cells and opened oligomers were purified by His-affinity purifications. (**b**) Native PAGE analysis of open and circular forms of GFP oligomers. (**c**) Analysis of discrete opened oligomers from dimer to pentadecamer by native PAGE. (**d**) *In-vitro* assemblies of opened GFP oligomers with the GFP 1–10 fragment. Linearly opened GFP trimer, tetramer and pentamer were reacted with excess GFP 1–10 and resulting protein assemblies were analysed in a native PAGE gel. (**e**) TEM images of opened GFP trimer and tetramer. (**f**) TEM images and schematic drawing of MBP-displayed opened GFP trimer. A possible protein arrangement in a representative TEM image (a copy of the first image) was suggested with dotted yellow circles. Scale bars, 10 nm.

Variation of a protein assembly process is highly desirable for more diverse spatial organization of functional proteins in nano-space. In addition to rather strained GFP polygons, we also constructed linearly opened and thereby more relaxed GFP oligomers. The GFP monomer was co-expressed with blocking CapGFP, where the GFP 11 strand was connected to the mature GFP (1–11; Fig. 4a). As circularization of GFP oligomers to form polygons is blocked by association of CapGFP, opened forms of GFP oligomers were generated. In addition, only CapGFPs were fused with a His-tag, and thereby linearly opened GFP oligomers were easily purified from a mixture of opened and closed (polygon) forms by Ni-affinity chromatography. Interestingly, opened GFP oligomers were more distinctly separated in a native gel than polygons (Fig. 4b). Moreover, oligomers with large sizes were more dominantly produced in open forms (Supplementary Fig. 14). As a result, we were able to isolate discrete linearly opened GFP oligomers up to 15 mer (Fig. 4c). Over 80 mg of opened GFP oligomers were obtained from 1 litre of *E. coli* culture, from which 4–6 mg of discrete oligomers from 3 mer to 10 mer could be prepared. For longer opened GFP oligomers (11 mer–15 mer), 1–2 mg of each pure oligomer could be still obtained.

Linear assembly process leaves an unreacted 11 strand of GFP monomer at one side of the oligomer (Fig. 4a), which can assemble with an additional GFP 1–10 fragment. To confirm the linearly opened structures of co-expressed GFP oligomers, we demonstrated *in-vitro* assembly of purified trimers, tetramers and pentamers with GFP 1–10, to form an additional GFP unit (Fig. 4d). Purified oligomer bands were completely shifted by the assembly, whereas GFP polygons were not affected by GFP 1–10 (Supplementary Fig. 15), indicating that highly pure open oligomers were prepared free from closed polygonal GFP oligomers. Linearly opened and thereby varied GFP arrangements of the oligomers were also visualized in TEM images of purified trimers and tetramers (Fig. 4e). For longer oligomers (>pentamer), however, many individual GFP monomers were not distinctly visualized and appeared to be gathered together in their TEM images (Supplementary Fig. 16). The data indicate the highly flexible nature of the linearly opened GFP oligomer. Rather disordered locations of fused MBP proteins on the linearly opened GFP trimer were also observed (Fig. 4f).

### Study of multivalency using functionalized GFP polygons

**Figure 5:**
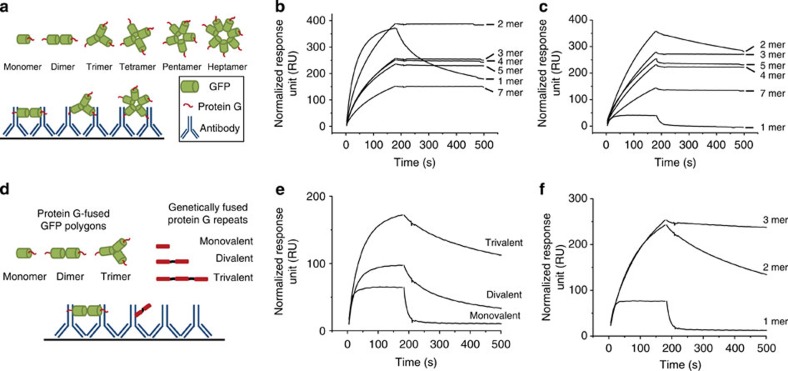
Multivalent interactions of protein G-functionalized GFP polygons. (**a**) Schematic representation of the multivalent interactions between surface-bound antibodies and protein G polygons. Multivalent protein G polygons (dimer to heptamer) and monomeric protein G-GFP were applied on the surface-bound antibodies. (**b**,**c**) SPR responses on the associations (180 s) and dissociations (320 s) of multivalent protein G polygons against human (**b**) or mouse (**c**) antibodies. Constant mass concentrations of protein G polygons (5 μg ml^−1^ for human and 10 μg ml^−1^ for mouse antibodies) were used to maintain constant concentrations of the protein G unit, regardless of the valency. (**d**) Schematic representation of the interactions of surface-bound antibodies with protein G polygons (mono-, di- and trimers) or with genetically fused protein G (mono-,di- and trivalent) repeats. (**e**,**f**) SPR responses on the associations (180 s) and dissociations (320 s) of genetically fused protein G repeats (**e**) or protein G polygons (**f**) against mouse antibodies. All binding curves were normalized by subtracting the reflective index changes on sample injections.

Multivalent interaction is an important strategy in nature to achieve highly enhanced but also dynamic bindings, in particular between biological systems^31^. For the study and use of this multivalency, systematic construction of multivalent ligand display scaffolds is required^32^,^33^. Although various chemically synthesized scaffolds were developed^33^,^34^,^35^, fully protein-based and monodisperse scaffolds with precisely controlled valency must be created for comprehensive study of multivalent protein–protein interactions. Here we employed our protein G-functionalized polygons (Fig. 3c) to provide experimental insight into avidity interactions between surface-bound antibodies and multivalent protein G (Fig. 5a and Supplementary Fig. 17). Protein G specifically interacts with the Fc region (one of constant domains) of an antibody. Human (Fig. 5b) and mouse (Fig. 5c) antibodies were immobilized on a surface plasmon resonance (SPR) sensor chip and purified protein G polygons (at constant monomer concentrations) were applied to the surfaces. Protein G binds more strongly to human antibodies than to mouse antibodies^36^. Binding sensorgrams of protein G monomer (1 mer) correlate well with relative binding affinities to both antibodies (Fig. 5b,c). Surface binding of protein G polygons (2 mer–7 mer) during oligomer injection (the first 180 s) was gradually decreased as the protein G valency increased, probably because the polygon concentration was proportionally lowered with the valency at constant monomer concentration. For human antibody (or protein G-binding antibody fragment, Supplementary Fig. 18), even dimeric protein G (2 mer) shows negligible dissociation (180 s–500 s) from the antibody surface (Fig. 5b). Against mouse antibody, however, more than three displayed protein G were required for similarly slow dissociations (Fig. 5c). Dissociation rate constants (*k*_off_) appeared to be more substantially influenced by the valency of protein G than association rate constants (*k*_on_). Simulated binding curves with constant *k*_on_ and varied *k*_off_ showed remarkable similarities with our experimental data (Supplementary Fig. 19). For instance, binding curves of mouse antibody to 1 mer, 2 mer and 4 mer of protein G polygons were nearly identical to kinetic binding models featuring *k*_off_ 10^−1^, 10^−3^ and 10^−5^ s^−1^, respectively, with a constant *k*_on_ 0.5 × 10^5^ M^−1^ s^−1^.

We next investigated the influence of the spatial arrangement of binding proteins on multivalent interactions. Protein G was genetically fused to form repeated protein G domains in a single chain (protein G repeats) and their binding to surface-bound mouse antibody was compared with that of protein G polygons (Fig. 5d). Divalent and trivalent protein G repeats provided stronger interactions to surface antibodies than a monovalent protein domain (Fig. 5e). Interestingly, however, slowed dissociation by increased valency was considerably less effective in protein G repeats than in polygons (Fig. 5e,f). More widely spaced protein G domains on GFP polygons will probably have a better chance to interact with multiple surface antibodies, whereas protein G repeats may mostly interact with a single antibody (Fig. 5d). Although multiple protein G domains (in both protein G repeats and polygons) statistically enhance binding/re-bindings to a single surface antibody, proper orientation and spatial arrangement of these binding domains (in polygons) are required for multi/multi protein G-antibody interactions (between multiple protein G and multiple antibodies). These multi/multi-interactions are probably responsible for slowed (especially negligible) dissociation of protein G polygons from surface antibodies. The results provide experimental evidence on the relative contributions of statistically enhanced interactions and multi/multi-interactions to enhanced affinities by multiple protein bindings^37^,^38^.

**Figure 6:**
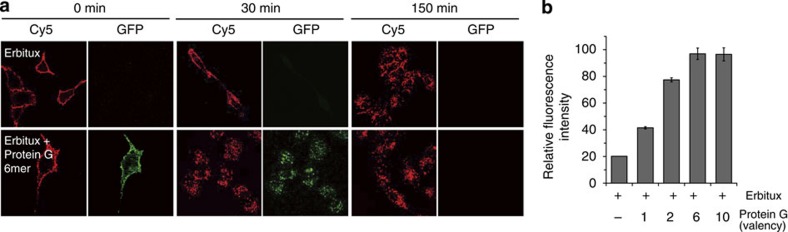
Receptor clustering and internalization by protein G-functionalized GFP polygons. (**a**) Confocal microscopy analysis of enhanced internalization of antibody–receptor clusters by protein G polygons. Cy5-labelled Erbitux (targeting EGFR) was treated to A549 cells with or without subsequent protein G hexamer treatment at 4 °C. After temperature change to 37 °C, receptor internalization was monitored by imaging Erbitux (Cy5) as well as protein G hexamer (GFP) at different time points. Cy5-Erbitux, red; Protein G-hexamer, green. (**b**) Flow cytometry analysis of antibody-mediated receptor internalization. A549 cells were treated with Cy5-Erbitux and subsequently protein G polygons with various valencies. Internalized levels of Cy5-Erbitux were quantified by flow cytometry. The error bars correspond to the s.e.m. of three independent measurements.

Cellular communications by cell surface receptors are also heavily governed by multivalent ligand–receptor interactions^33^,^39^. Ligand-induced clustering of receptors is one of the key mechanisms underlying cellular signal transduction, and thereby various multivalent ligand systems have been used to study and control cellular signalling^40^,^41^,^42^. We lastly exploited multivalent protein G polygons to control antibody-mediated clustering of epidermal growth factor receptor (EGFR) on cell surfaces. Antibody drug (such as Erbitux) treatment is known to induce EGFR downregulation, which is mediated by receptor internalization without activation of downstream signalling^43^. Several reports proposed that the rate of EGFR internalization is affected by the size of receptor clusters^44^,^45^,^46^. We investigated whether discrete protein G polygons can systemically manipulate the level of antibody–EGFR clusters and subsequently receptor internalization. A549 cells (human lung adenocarcinoma) were sequentially treated with dye (Cy5)-labelled Erbitux (anti-EGFR monoclonal antibody) and multivalent protein G polygons at 4 °C. Cells were then incubated at 37 °C to initiate receptor internalization and cellular distributions of Erbitux (red) and protein G polygons (green) were monitored by confocal microscopy (Fig. 6a). The rate of Erbitux internalization was significantly increased by protein G hexamer (6 mer). Internalization of Erbitux clustered by protein G hexamer was observed in 30 min, whereas Erbitux alone was slowly internalized within 150 min. Internalized GFP signals disappeared at 150 min, possibly due to protein degradation after internalization^43^. Flow cytometry measurement indicated that intracellular accumulation of Erbitux was augmented as the valency of protein G polygons increased (Fig. 6b). Erbitux internalization was also slightly enhanced by monomeric protein G, although nonspecific binding of protein G polygons was not observed on cell surfaces (Supplementary Figs 20 and 21). Erbitux accumulation was saturated after prolonged incubation (3 h), regardless of added protein G polygons (Supplementary Fig. 22). These results suggest that our polygonal scaffold can stimulate antibody-mediated clustering of EGFR on cell surfaces and accelerate the internalization of these receptor clusters.

## Discussion

We have shown a new strategy to construct monodisperse supramolecular protein assemblies with defined structures and varied numbers of protein-building blocks. GFP monomers were designed to be self-assembled in cells for facile production of highly stable GFP oligomers (both polygonal and open forms). Oligonucleotide-like charge engineering on the oligomers allowed isolation of monodisperse GFP assemblies from dimer to up to 15 mer in native conditions, which is essential to maintain protein functions. Monodispersity of the subsequently isolated species of the oligomers also made it possible to determine nanoscale arrangements of protein units in the oligomers by a direct TEM analysis. We envision that our approach will be applicable to other protein-assembling methods to produce many more discrete protein assemblies with diverse structures and functions. Our discrete protein oligomers can also serve as new building blocks (or tiles) for the construction of even higher-order protein structures and as new templates for the assembly of high-order nanostructures.

We have also demonstrated accurate control of multivalent protein display on our protein polygons with exact numbers, arrangements and orientations. Our protein scaffolds will offer unprecedented tools to design experiments to understand principles of multivalent protein interactions, as we evaluated the effects of nanoscale spatial arrangement as well as the valency of binding proteins on multivalent binding kinetics. More studies by using diverse protein scaffolds including our linearly opened oligomer (with different shapes, spacing and rigidity) and a wide set of binding proteins will follow. Multivalency is also a key principle in many biological processes such as viral entry, cell–cell communications, immune responses and cellular phase separations^31^,^47^,^48^. The use of discrete protein nano-scaffolds developed in the present study will be also highly beneficial for future understanding and control of these processes.

## Methods

### Expression and purification of GFP oligomers

The genes encoding various GFP monomers were cloned into pET-28a (Novagen) expression vector. For the construction of opened GFP oligomers, the genes for both CapGFP and GFP monomer were cloned into MCS1 (multiple cloning site 1) and MCS2 of the dual gene expression vector pACYDuet-1 (Novagen), respectively. Protein sequences of GFP monomers are provided in Supplementary Note 1. *E.coli* BL21 (DE3) cells harbouring the appropriate vectors were grown at 37 °C until OD_600_=0.6 and proteins were induced with 1 mM isopropyl-1-thio-β-D-galactoside and then incubated at 20 °C overnight. The induced cells were collected by centrifugation and lysed by sonication into a buffer appropriate for subsequent affinity chromatography. His_6_-tagged GFP oligomers were purified from soluble protein fractions of cell lysates using Ni-NTA (Qiagen). Oligomers were stored at 4 °C for 1–2 months. Oligomer concentrations were determined by both Bradford and BCA assays. Fluorescence images of the oligomers in PAGE gels were acquired by Chemi-doc (Biorad).

### Preparation of discrete GFP oligomers

A mixture of GFP oligomers was separated on a 6% native PAGE gel (HSI SG 600 Series, Hoefer Scientific Instruments, 16 × 18 cm). Electrophoresis was performed at 280 V for 150–180 min. After electrophoresis, fluorescent bands of the oligomers were visualized using ultraviolet light and excised. Sliced gels were introduced into a dialysis membrane (Spectrum Labs, MWCO 3,500) and filled with the electrode running buffer (25 mM Tris base and 192 mM glycine). The oligomers were eluted from the gels at 100 V for 40–60 min in a horizontal gel electrophoresis apparatus, performed at 4 °C with a pre-chilled buffer. The eluted fractions containing protein oligomers were collected and NaCl was added to a final concentration of 125 mM. For further applications, eluted oligomers were concentrated by polyethylene glycol 8,000 (LPS solution). The oligomer solution was placed in a dialysis membrane and put on polyethylene glycol 8,000 powder. Oligomers were generally concentrated to >0.5 mg ml^−1^. When necessary, the oligomer solutions were additionally dialysed against PBS.

### Size-exclusion chromatography

GFP oligomers were injected onto a Superdex 200 column (10/300 GL, GE Healthcare) and run at 0.5 ml min^−1^ with an elution buffer (500 mM NaCl, 50 mM Tris pH 8.0 or PBS). Protein elution was monitored at 280 nm. For analysis of discrete GFP polygons, absorbance spectra were monitored at 488 nm and normalized on their maximum peaks.

### Negative-stain electron microscopy

GFP oligomers were adsorbed to carbon grids and negatively stained with 0.75% uranyl formate. Electron micrographs were acquired with a 4 K × 4 K Eagle HS CCD (charge-coupled device) camera (FEI) on a Tecnai T120 microscope (FEI) at 120 kV. Images were taken at a magnification of × 67,000 and defocus settings ranging from −1.4 to −1 μm.

### SPR analysis

SPR experiments were performed in a Biacore 3000 instrument using dextran CM5 gold chips (GE Healthcare), using a PBS buffer as a running solution. Human immunoglobulin G (IgG) (Sigma 12511, 2,000 response unit, RU) and monoclonal mouse IgG1 (R&D System 71903, 4,000 RU) were immobilized on sensor chips using EDC/NHS amine coupling. Excess reactive groups were blocked with 1 M ethanolamine (pH 8.5). Protein G polygons were treated to the antibody-bound surfaces (5 μg ml^−1^ for human and 10 μg ml^−1^ for mouse antibodies) at a flow rate of 30 μl min^−1^. Binding properties of protein G polygons were analysed by monitoring association and dissociation phases for 180 and 320 s, respectively. After each measurement, the chip surface was regenerated using 10 μl injections of 10 mM NaOH, to remove bound protein G polygons before the next measurement. Binding curves were normalized by subtracting the reflective index changes on sample injections. For a comparative analysis of genetically fused protein G repeats (mono-, di- and trivalent) and protein G polygons (1-, 2- and 3 mer), monoclonal mouse IgG1 (Santa Cruz sc-138) was immobilized to reach 3,000 RU on a CM5 sensor chip. Both protein G constructs were treated to the same surface with the same monomer (or monovalent) concentration (6 μg ml^−1^ of genetically fused protein G and 30 μg ml^−1^ protein G polygons) at a flow rate of 30 μl min^−1^.

### Antibody-mediated receptor internalization assays

Erbitux (a kind gift from Dr Myung Kyu Lee at KRIBB, Daejoen, Korea) was labelled with Cy5 by reacting with Cy5 mono NHS ester (GE Healthcare PA15100). A549 cells were treated with a Cy5-Erbitux (10 μg ml^−1^) at 4 °C for 30 min. Unbound Erbitux was washed away and protein G polygons (10 μg ml^−1^) were subsequently treated, also at 4 °C for 30 min. Erbitux alone or Erbitux–protein G-bounded cells were incubated for 0, 30 and 150 min at 37 °C, to initiate receptor internalization. Thereafter, cells were washed and fixed with 4% paraformaldehyde and fluorescence images were obtained by using confocal microscopy (Carl Zeiss). To quantify internalized Erbitux–EGF receptor clusters, antibody or antibody–protein G-bounded cells were incubated for 10 min at 37 °C and then washed with pre-chilled PBS. Cell surface-bound antibodies were removed by acid stripping, followed by trypsinization and fixation with 4% paraformaldehyde. Internalized Cy5-Erbitux was analysed by flow cytometry (BD Biosciences FACSAria).

## Additional information

**How to cite this article**: Kim, Y. E. *et al*. Green fluorescent protein nanopolygons as monodisperse supramolecular assemblies of functional proteins with defined valency. *Nat. Commun.* 6:7134 doi: 10.1038/ncomms8134 (2015).

## Supplementary Material

Supplementary Information Supplementary Figures 1-22, Supplementary Tables 1-2 and Supplementary Note 1
