# Supplementary material for: Green fluorescent protein nanopolygons as monodisperse supramolecular assemblies of functional proteins with defined valency
Source: Nat Commun. 2015 May 14;6:7134. doi: 10.1038/ncomms8134 (PMC4479010; doi:10.1038/ncomms8134)
Supplement: Supplementary Information — Supplementary Figures 1-22, Supplementary Tables 1-2 and Supplementary Note 1 [file ncomms8134-s1.pdf]

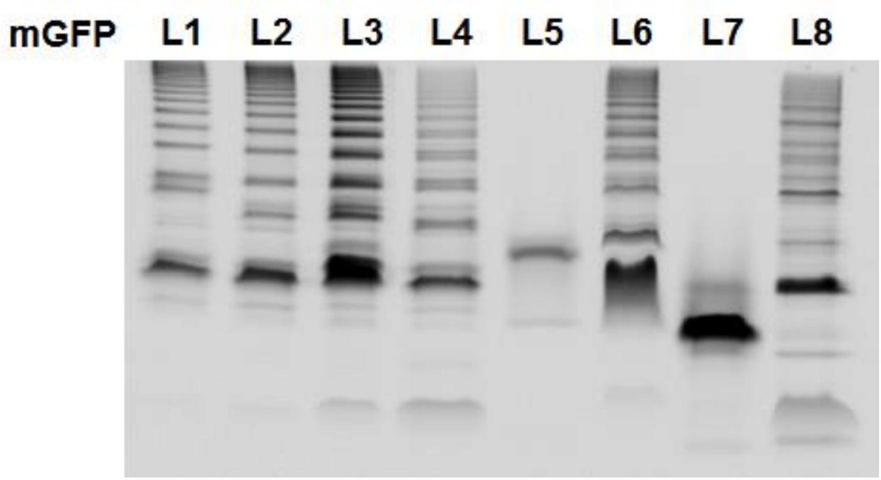

Supplementary Figure 1. SDS-PAGE analysis of GFP oligomer variants with different linkers. Oligomer mixtures were applied to a PAGE gel containing 0.1% SDS without boiling. The gel was analyzed by a fluorescent image analyzer with 470 nm-excitation and 530 nm-emission filters.

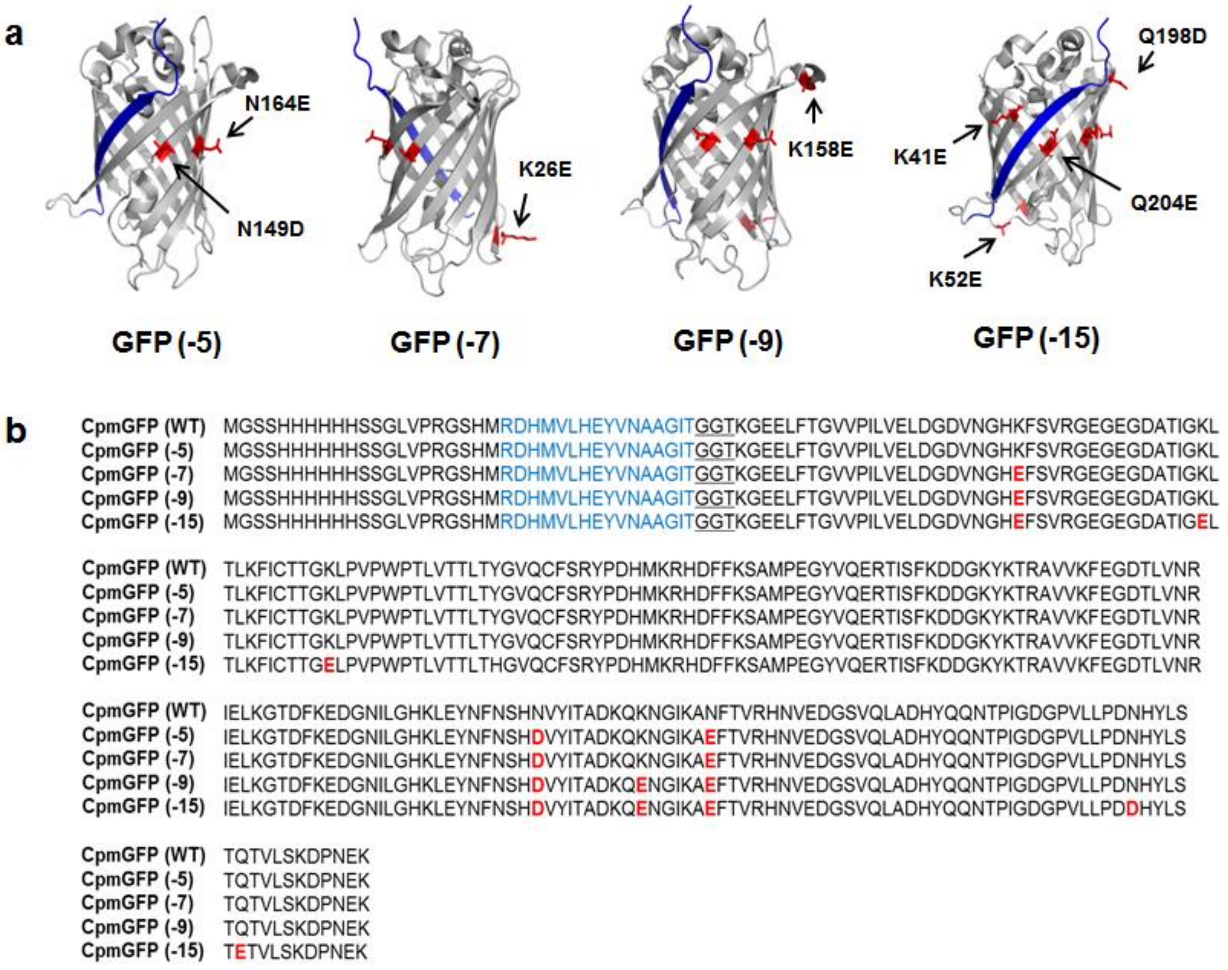

Supplementary Figure 2. Design of the charge variants of GFP monomer. a, Ribbon cartoon diagrams of the charge variants of GFP monomer. The GFP 11 strand is shown in blue. Substituted residues are shown in red, and mutations are indicated with arrows. **b,** Protein sequences of GFP monomer with net charges of -5, -7, -9 and -15. Blue, GFP 11; underlined, peptide linker; red, mutated residues.

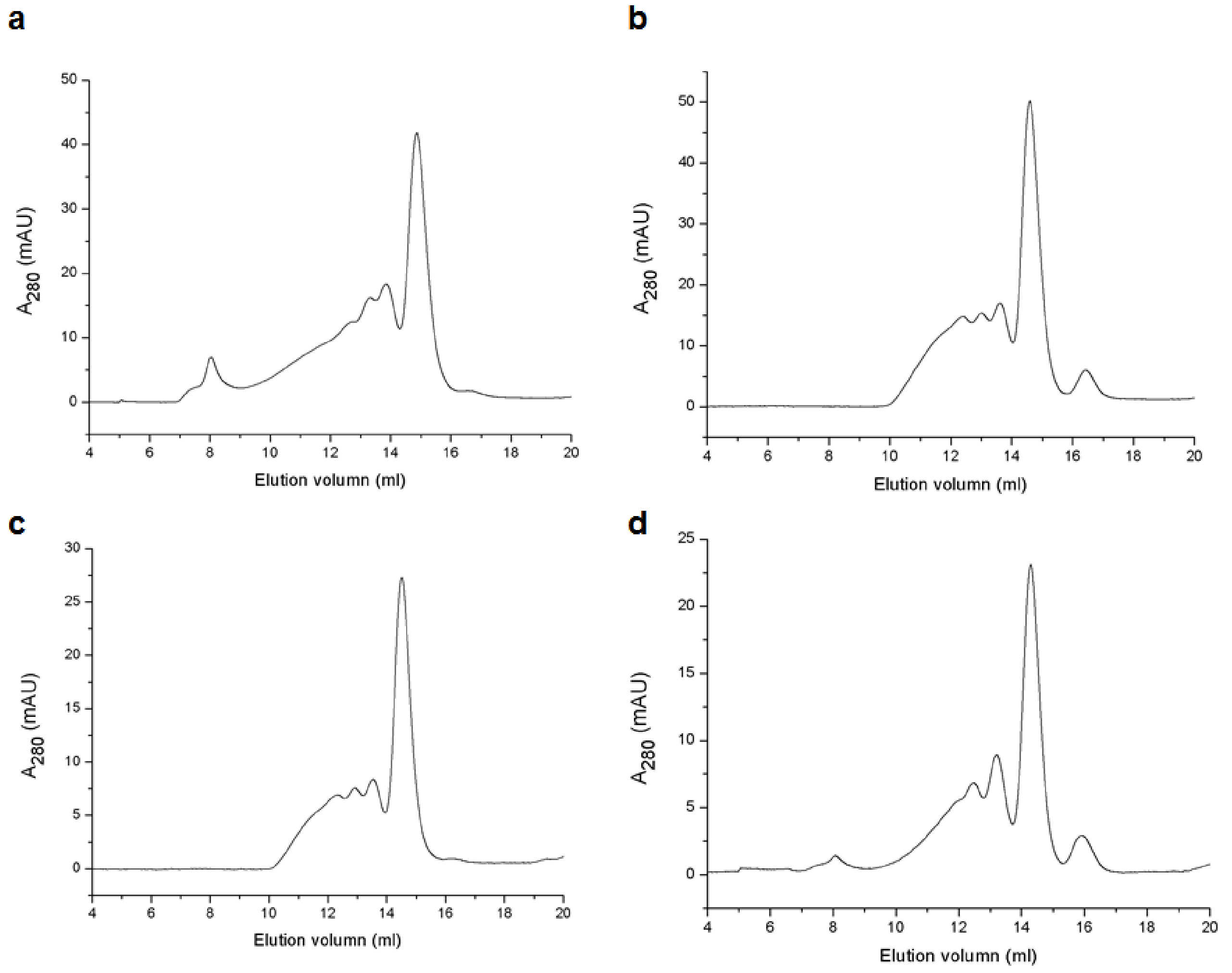

Supplementary Figure 3. SEC analysis of GFP oligomer charge variants. GFP oligomers with net charges of -5 (**a**), -7 (**b**), -9 (**c**) and -15 (**d**) were analyzed using a superdex 200 column (10/300 GL).

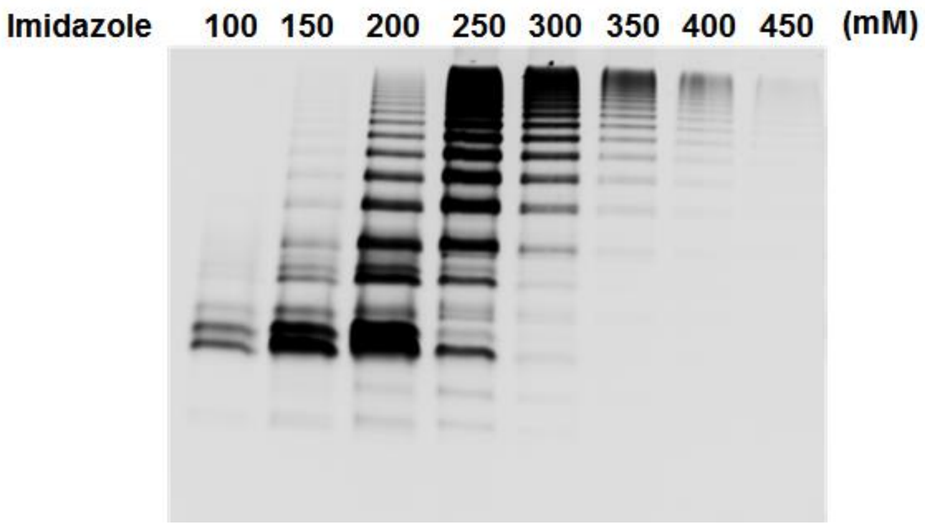

Supplementary Figure 4. Partial purification of GFP oligomers by affinity chromatography. GFP oligomers (net charge of -3) were eluted from a Ni-chelating column with elution solutions containing imidazole from 100 mM to 450 mM. Samples (without boiling) were analyzed by SDS-PAGE. The gel was analyzed by a fluorescent image analyzer with 470 nm-excitation and 530 nm-emission filters.

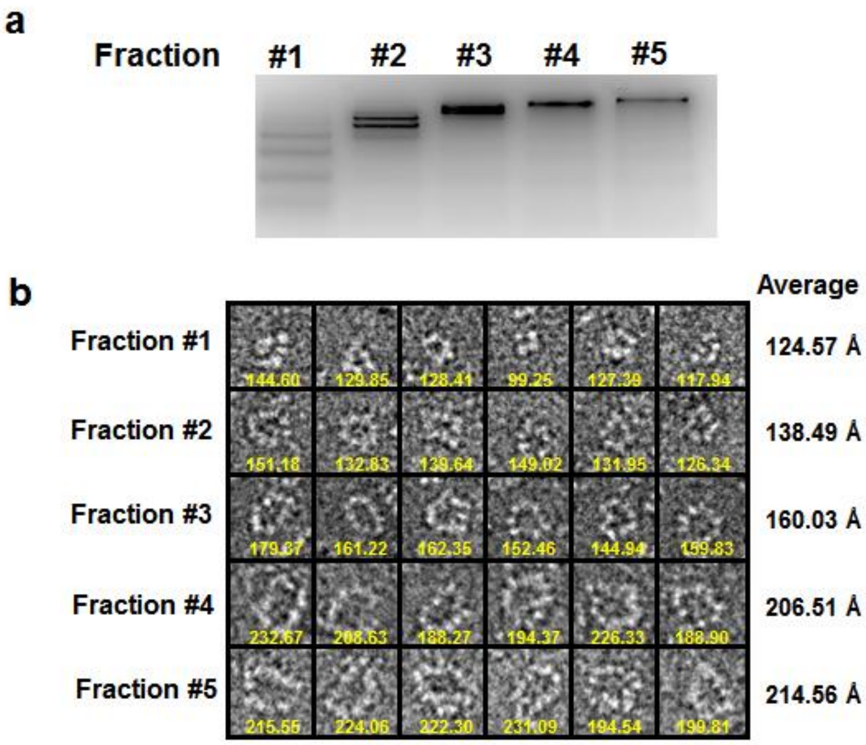

Supplementary Figure 5. TEM images of partially purified GFP polygons with a net charge of -3 (wild type). a, Mixtures of GFP polygons (net charge -3, wild type) were partially purified by the electro-elution method and analyzed by SDS-PAGE (without boiling). **b,** TEM images of purified GFP polygons from fraction #1 to fraction #5. For fractions #4 and #5, polygonal GFP oligomers with more than 10 GFP monomers were also observed.

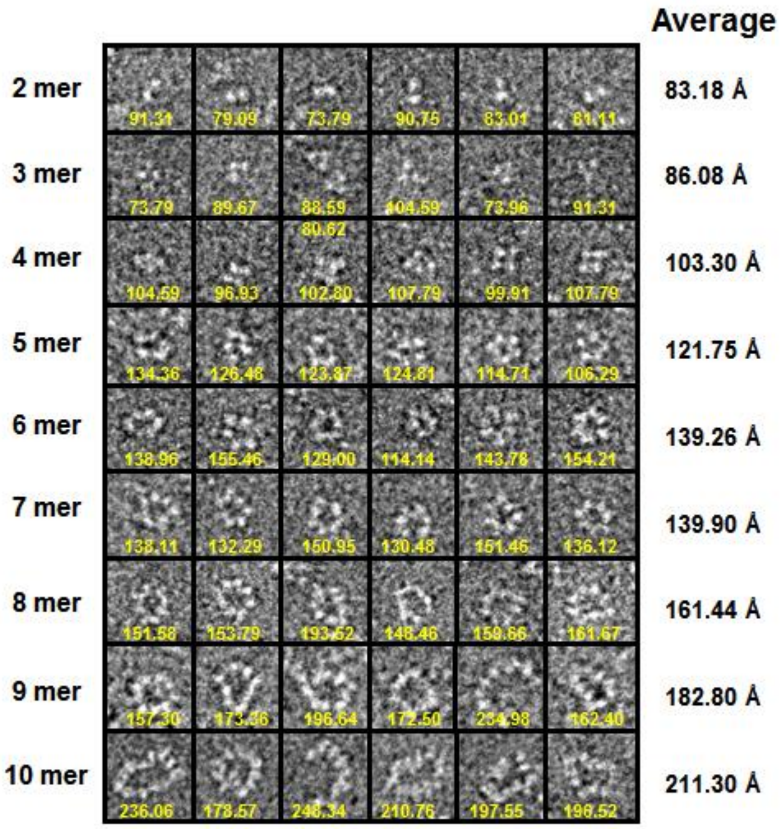

Supplementary Figure 6. Direct size measurement of discrete GFP polygons from dimer to decamer based on TEM images.

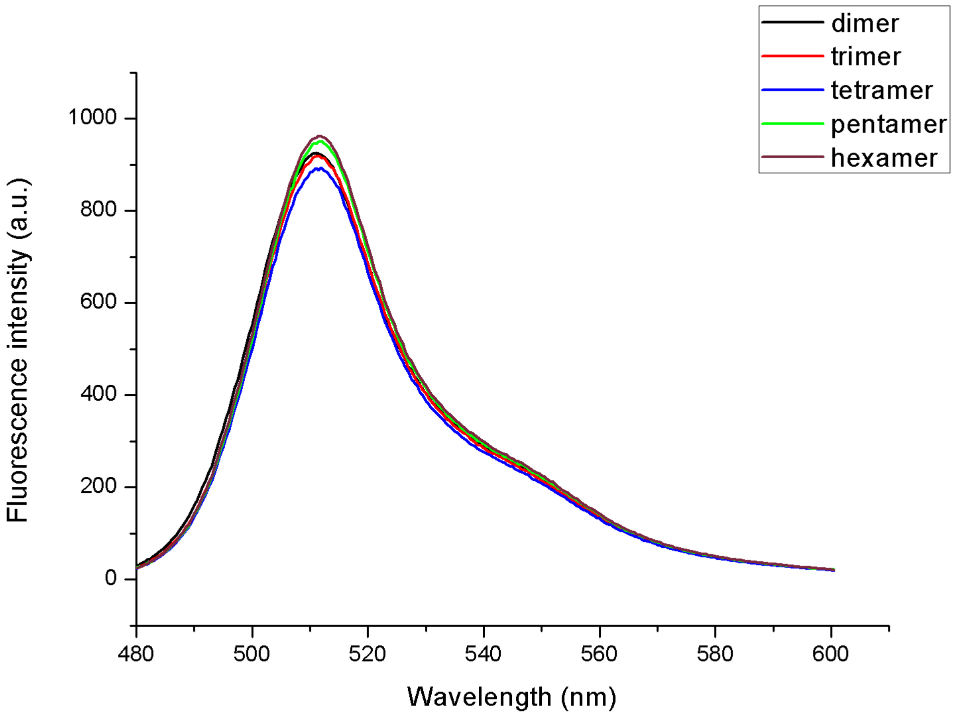

Supplementary Figure 7. Fluorescence emission spectra of di-, tri-, tetra-, penta- and hexamer of GFP polygons. Fluorescence emission profiles of GFP polygons with various oligomeric states (constant monomer concentration) were monitored with excitation at 460 nm.

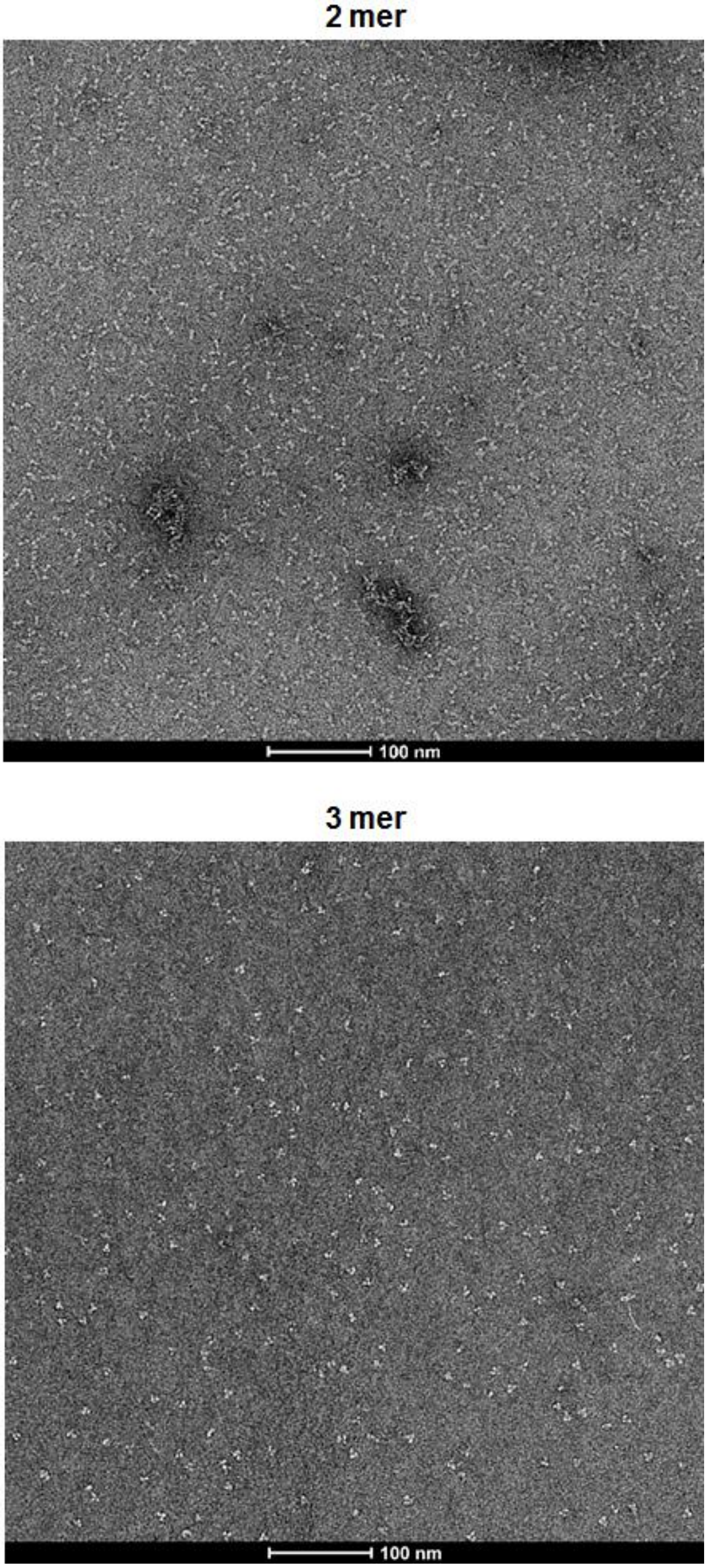

Supplementary Figure 8. Representative TEM images of GFP polygons, dimer (top) and trimer (bottom).

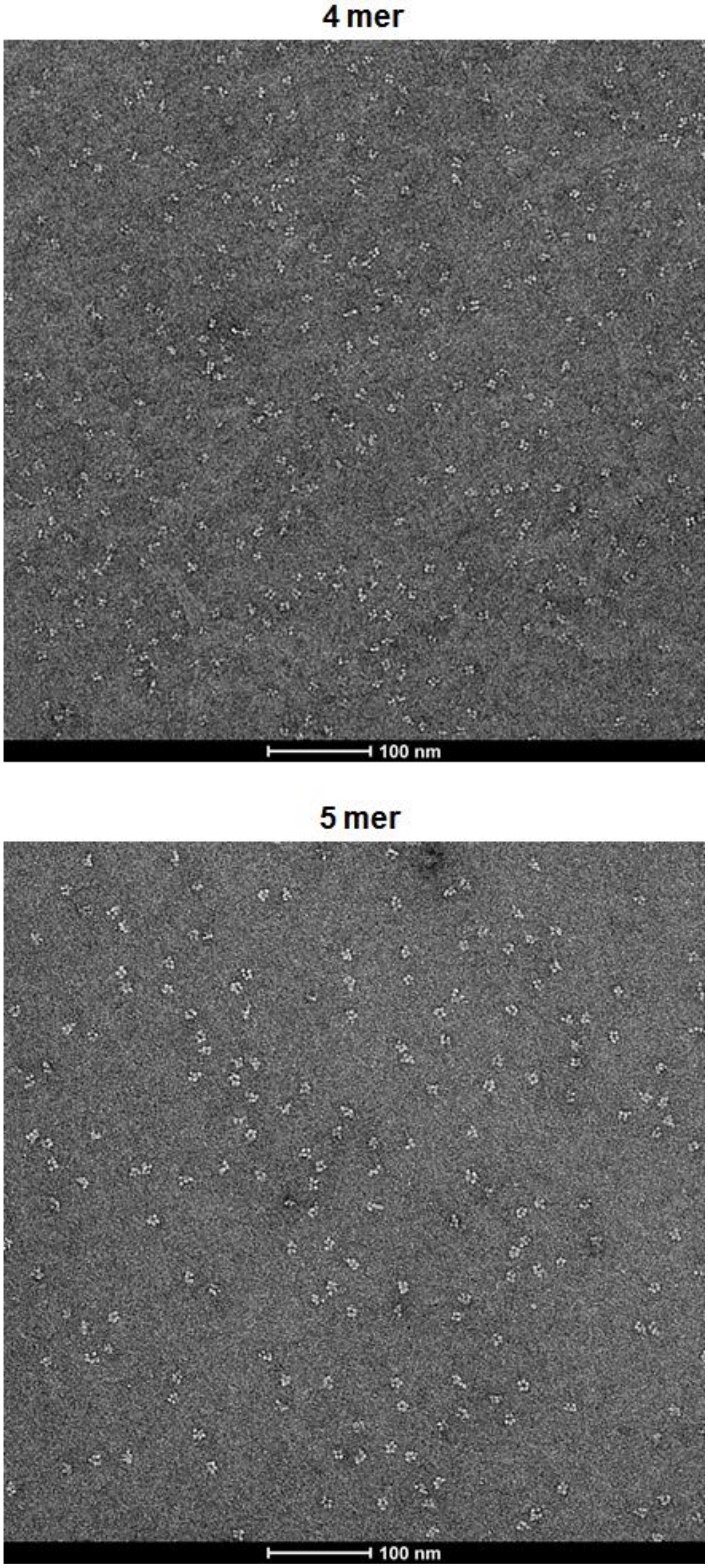

Supplementary Figure 9. Representative TEM images of GFP polygons, tetramer (top) and pentamer (bottom).

6 mer

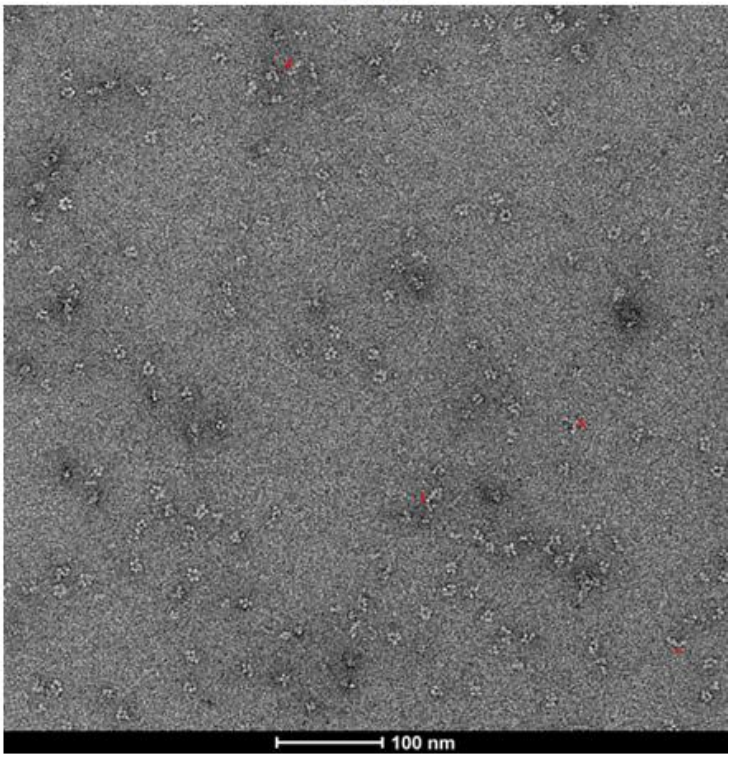

7 mer

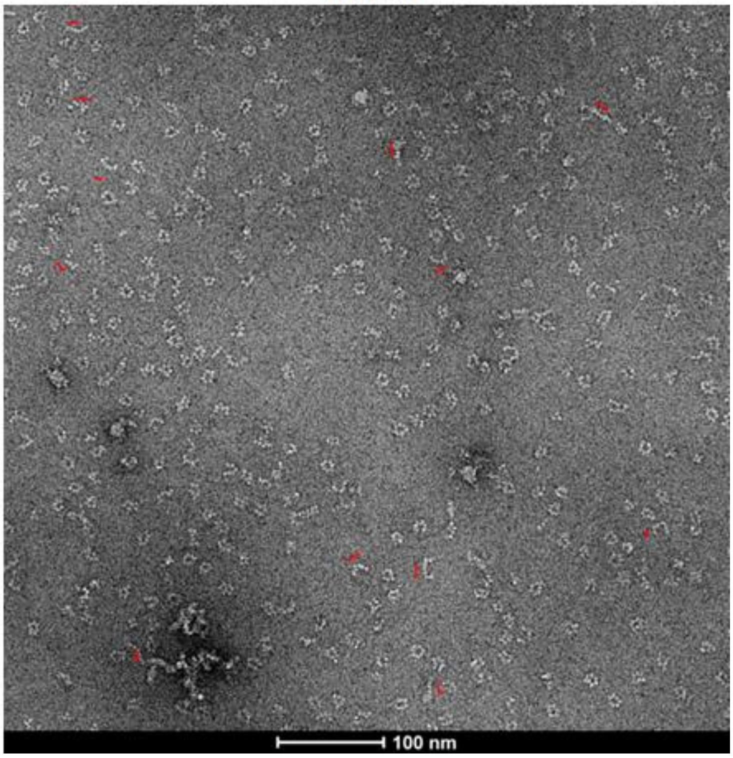

Supplementary Figure 10. Representative TEM images of GFP polygons, hexamer (top) and heptamer (bottom). GFP oligomers with rather linearly opened GFP arrangements are indicated with red lines.

8 mer

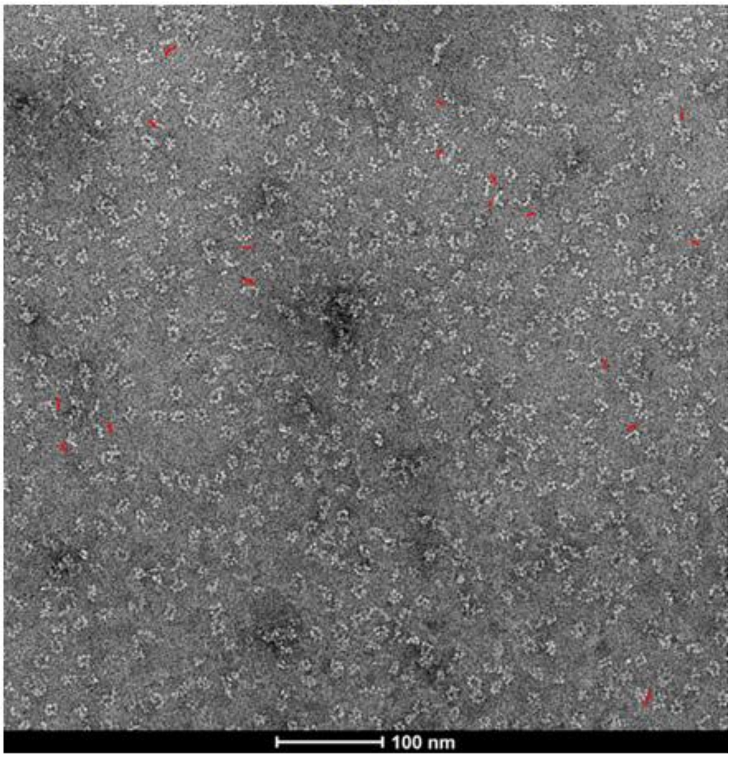

9 mer

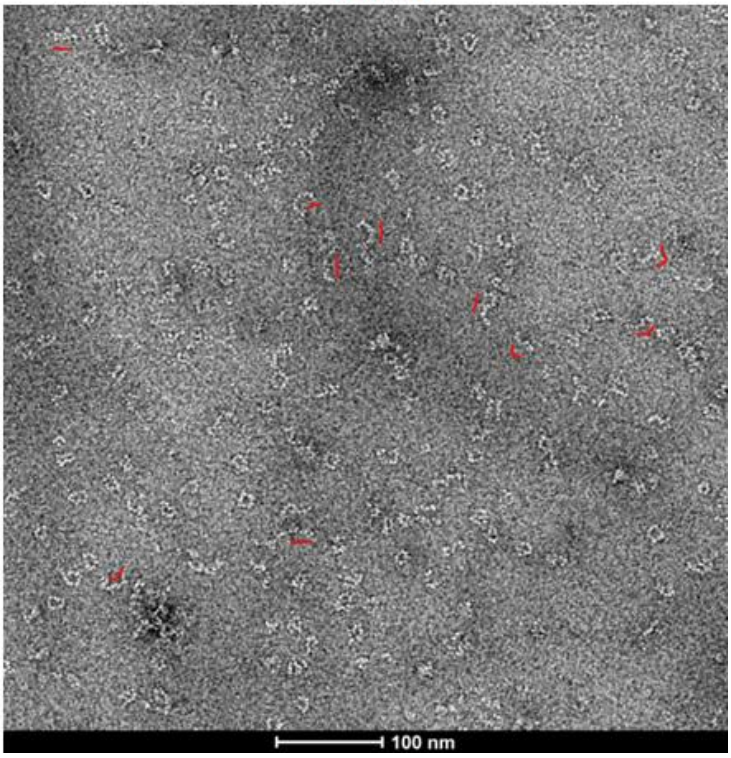

Supplementary Figure 11. Representative TEM images of GFP polygons, octamer (top) and nonamer (bottom). GFP oligomers with rather linearly opened GFP arrangements are indicated with red lines.

10 mer

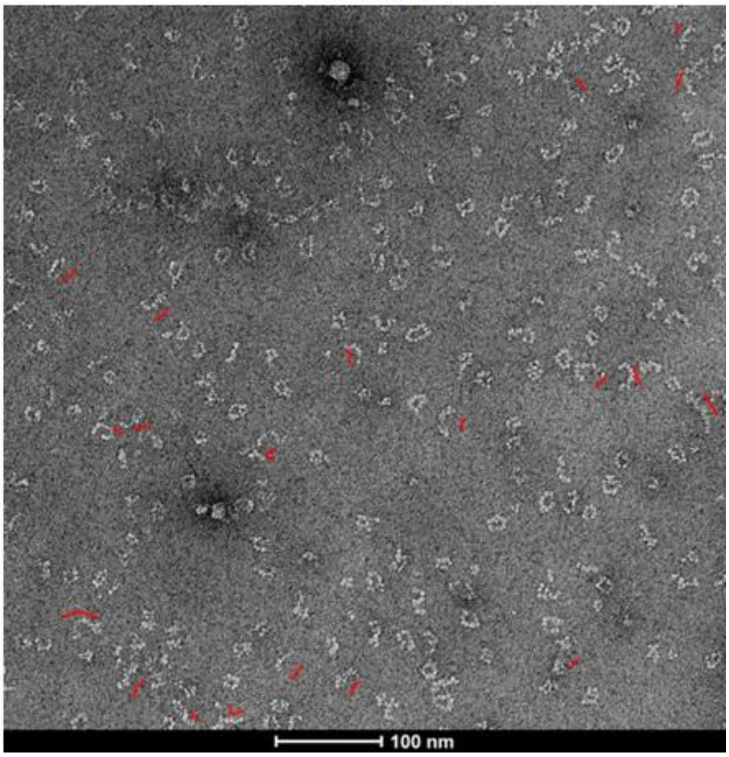

		6 mer		7 mer		8 mer		9 mer		10 mer	
Linear	Circular	4	140	12	250	17	360	10	162	20	191
Relative ratio		2.8%	97.2%	4.6%	95.4%	4.5%	95.5%	5.8%	94.2%	9.5%	90.5%

Supplementary Figure 12. Representative TEM images of GFP polygon decamer. GFP oligomers with rather linearly opened GFP arrangements are indicated with red lines. Intact polygonal GFP oligomers and (possibly) opened linear GFP oligomers are counted and summarized in the table. Scale bars, 100 nm.

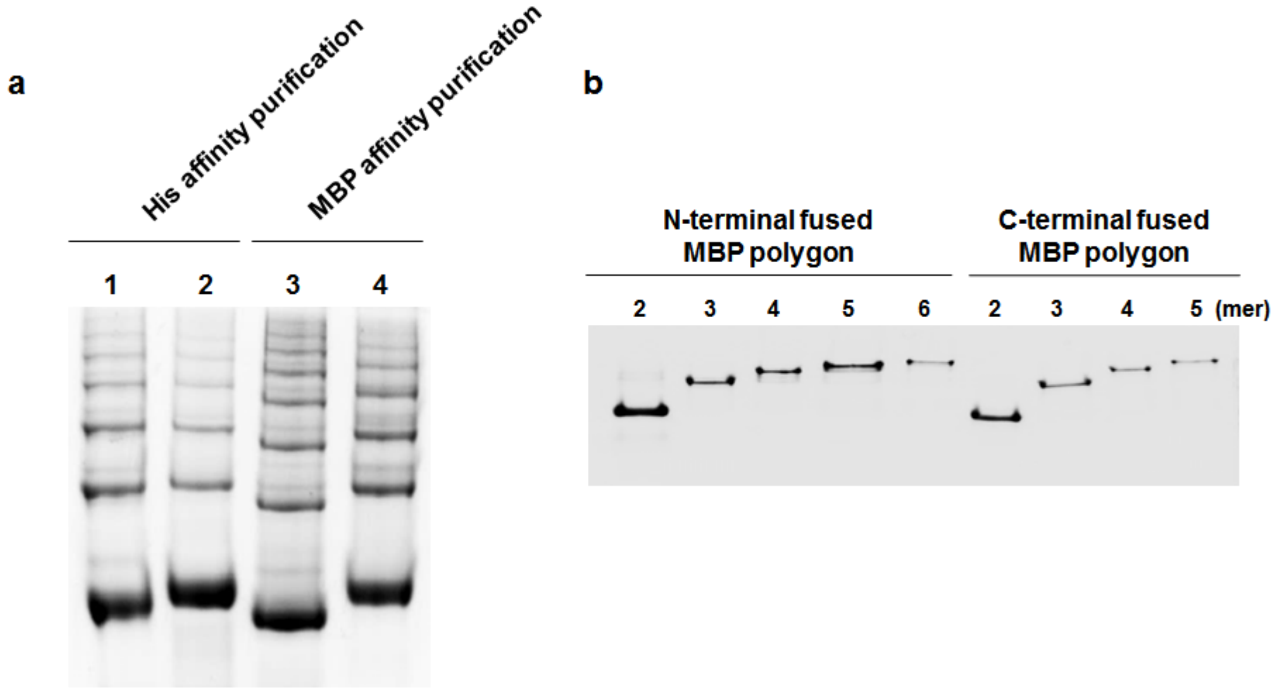

Supplementary Figure 13. Multivalent display of functional MBP on GFP polygons. a, Native-PAGE analysis of N- (lane 1 and lane 3) and C-terminal fused MBP polygons (lane 2 and lane 4), purified by His-affinity purification or MBP affinity purification. The gel was analyzed by a fluorescent image analyzer with 470 nm-excitation and 530 nm-emission filters. **b,** Native-PAGE analysis of discrete N- and C-terminal fused MBP polygons. Weak protein bands between major MBP-fused GFP polygon bands in Fig. S9a may be those of GFP polygons without some fused MBP proteins (likely one or two MBP), due to non-specific cleavage during protein expression. In the present study, however, the portion of protein assemblies without fused proteins can be minimized by the native gel-based protein purification process.

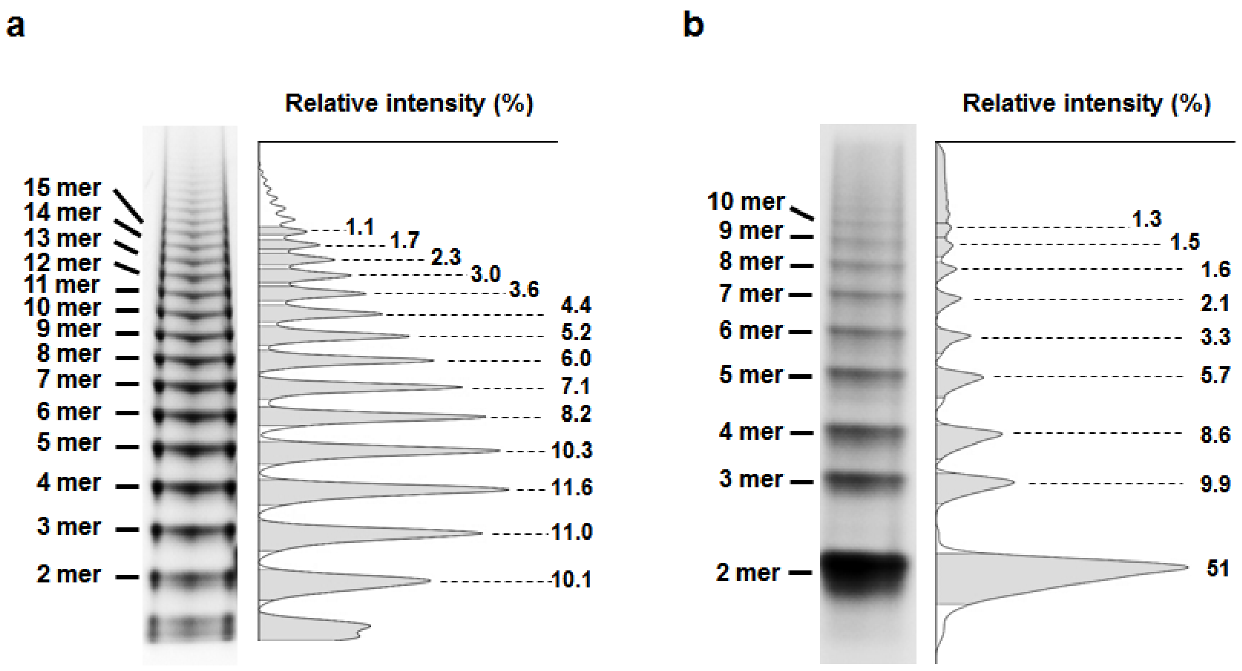

Supplementary Figure 14. Size distributions of GFP oligomers on native-PAGE. Relative intensities of GFP oligomer bands were analyzed by Image 4.1 software. **a,** Open form of oligomers **b,** Circular form of oligomers.

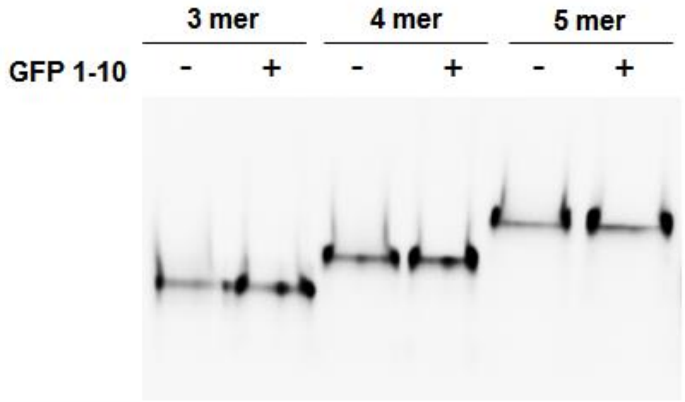

Supplementary Figure 15. *In vitro* assemblies of GFP polygons with the GFP 1-10 fragment. GFP polygons (trimer, tetramer, and pentamer) were reacted with excess GFP 1-10, and resulting protein assemblies were analyzed in a native-PAGE gel.

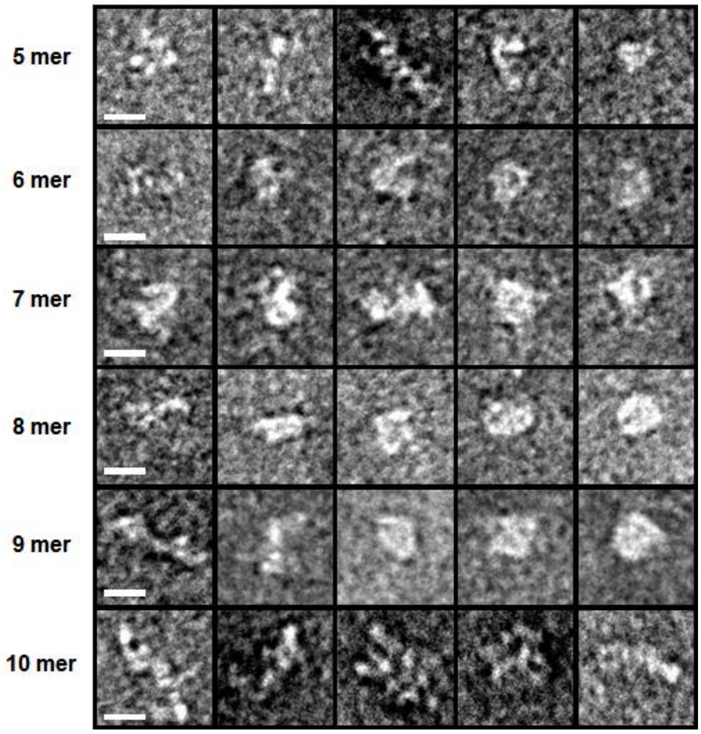

Supplementary Figure 16. Representative TEM images of opened GFP oligomers from pentamer to decamer. Scale bars, 10 nm.

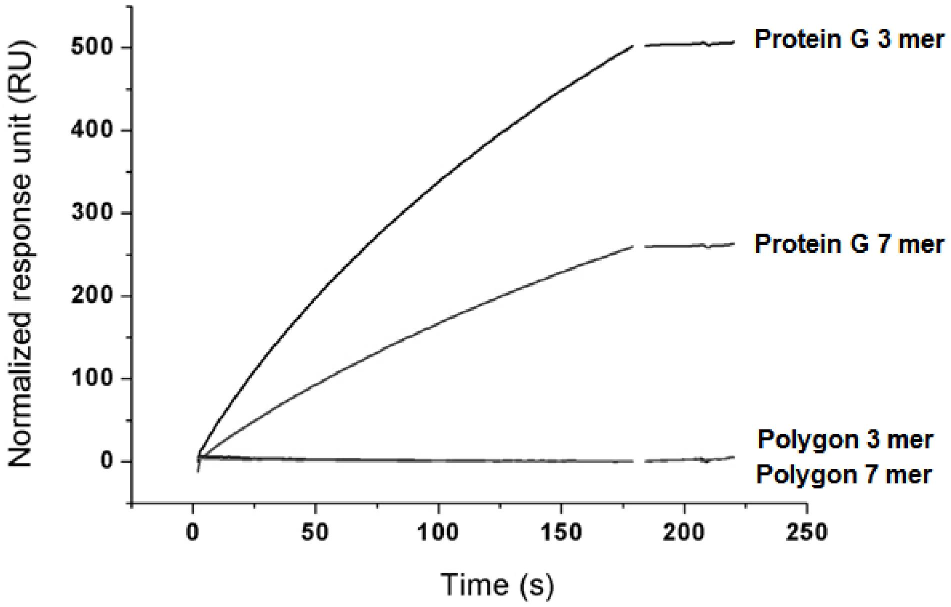

Supplementary Figure 17. Specific interaction of protein G-fused GFP polygons to surface-bound antibodies. A SPR sensor chip surface was covered with mouse IgG1 (4000 RU), and GFP polygons (Polygon 3 mer & 7 mer) as well as protein G-fused GFP polygons (Protein G 3 mer & 7 mer) were applied at constant monomer concentration (10 μg ml^{-1}). Binding curves were normalized by subtracting the reflective index changes upon sample injections.

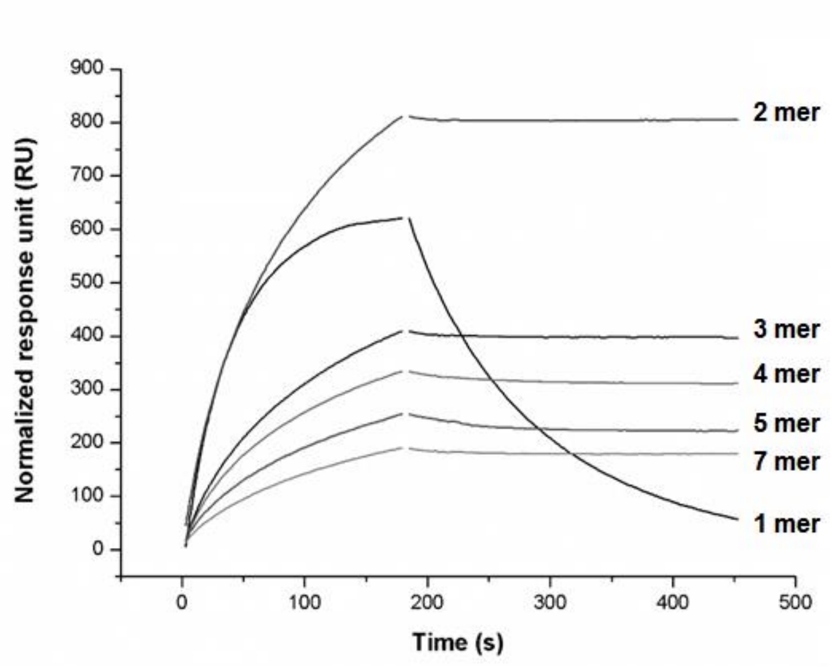

Supplementary Figure 18. SPR responses upon multivalent protein G polygon binding to human Fc domain. A SPR sensor chip surface was covered with recombinant human Fc domain (15000 RU), and protein G-fused polygons were applied at constant monomer concentration (5 μg ml^{-1}). Binding curves were normalized by subtracting the reflective index changes upon sample injections. Here recombinant human Fc protein was employed, whereas human IgG mixtures (isolated directly from human serum) were used in Figure 5b in the main text. More homogeneous interaction between Fc domain and protein G was expected, and binding curves of Fc domain were more similar to the simulated curves (Supplementary Figure 19a) than those of human IgG.

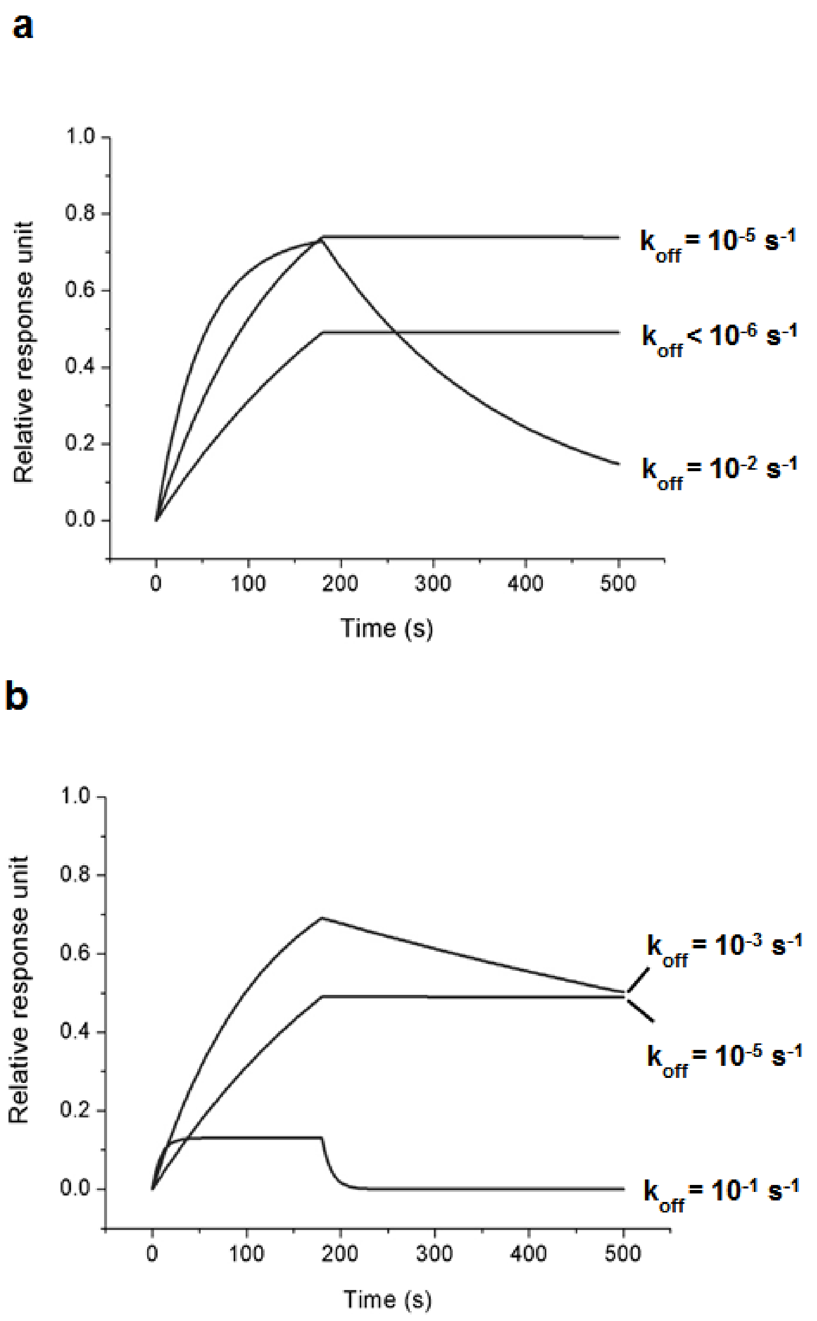

Supplementary Figure 19. Simulated SPR binding curves of multivalent protein G polygons with surface-bound human IgG (a) and mouse IgG (b). a, 150 nM of protein G was applied to surface-bound human IgG. Association (180 sec) and dissociation (320 sec) phases were simulated with an association constant (k_{on}) 1 × 10^5 ($M^{-1}s^{-1}$) and dissociation constants (k_{off}) 10^{-2}, 10^{-5}, and 10^{-6} (s^{-1}). **b,** 300 nM of protein G was applied to surface-bound mouse IgG1. Association (180 sec) and dissociation (320 sec) phases were simulated with an association constant (k_{on}) 0.5 × 10^5 ($M^{-1}s^{-1}$) and dissociation constants (k_{off}) 10^{-1}, 10^{-3}, and 10^{-5} (s^{-1}). The units are relative response units (RU/RU_{max}). The simulation equation for association curves is [relative response units (RU/RU_{max}) =

$\frac{C}{C+koff/kon}(1-e^{-(kon\cdot C+koff)t})$]. The simulation equation for dissociation curves is [relative response units (RU/RU_{max}) = (RU_{eq}/RU_{max}) $\cdot e^{-koff\cdot t}$]. In these equations, terminology definitions are as follow: RU_{max}: maximum responsive unit, RU_{eq}: equilibrated responsive unit, C: polygon concentration (M), $koff$: dissociation rate constants (s^{-1}), kon: association rate constants ($M^{-1}s^{-1}$), t: time (sec).

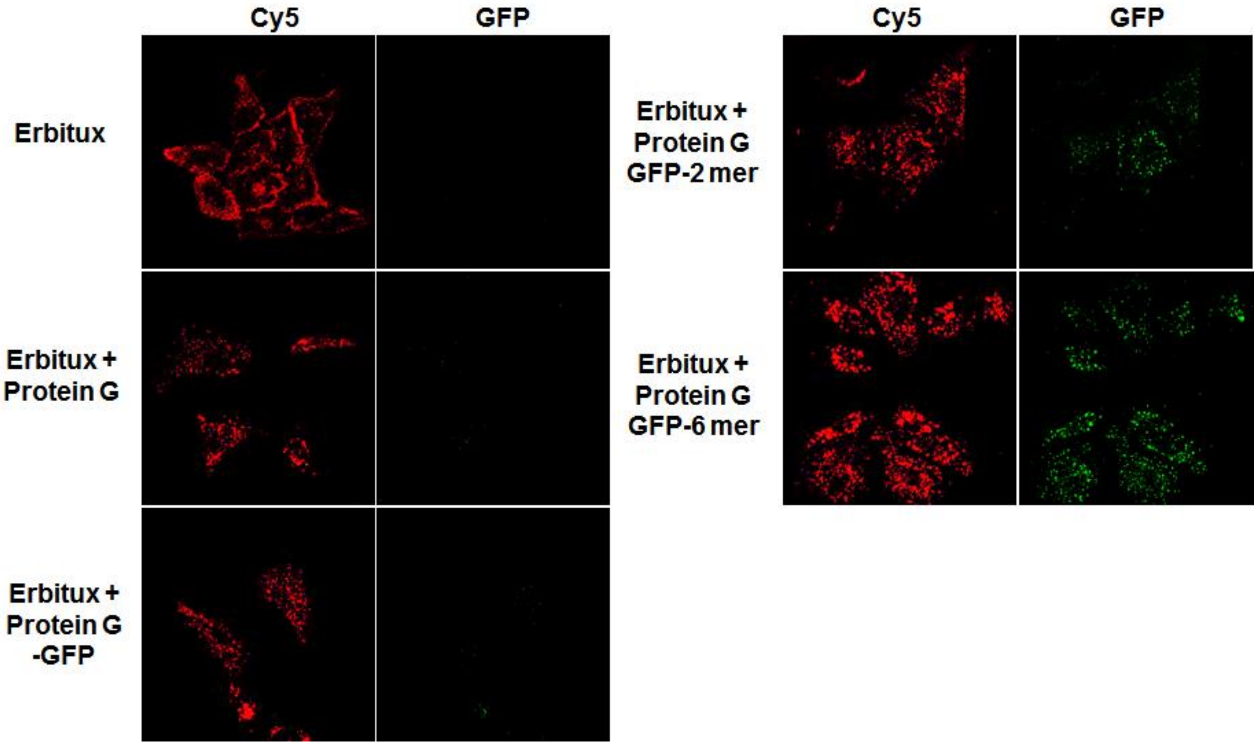

Supplementary Figure 20. Confocal microscopy analysis of internalization of antibody-receptor clusters by protein G polygons with various valency. A549 cells were sequentially treated with Cy5-Erbitux (10 μg ml^{-1}) and protein G-fused GFP polygons (10 μg ml^{-1}) with various valency or free protein G (10 μg ml^{-1}). Erbitux alone or Erbitux-protein G bounded cells were incubated for 30 min at 37 ℃, and receptor internalization was monitored by confocal microscopy. Cy5-Erbitux, red; protein G-polygon, green.

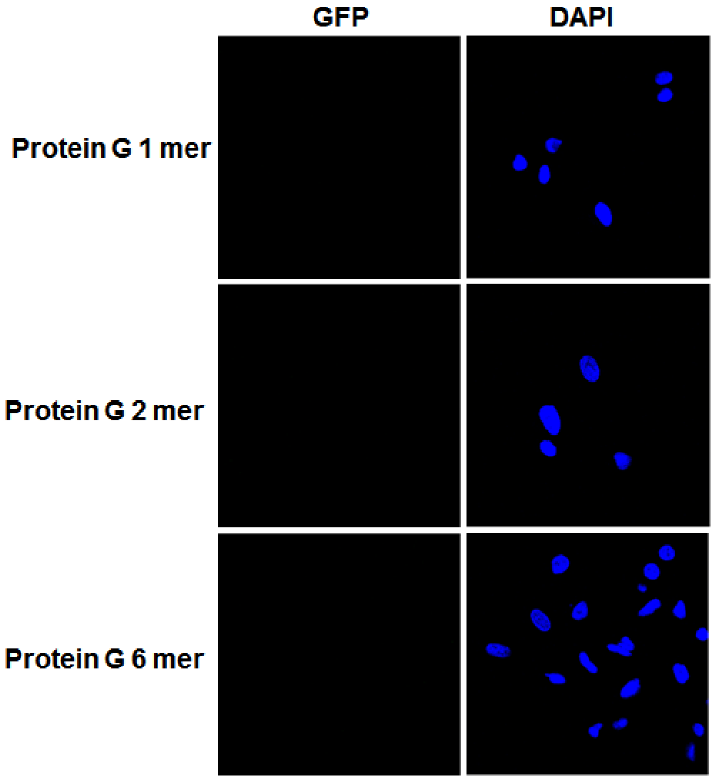

Supplementary Figure 21. Non-specific interactions of protein G polygons on cell surfaces. A549 cells were treated with protein G polygons (10 μg ml^{-1}) and incubated for 30 min at 37 °C. Nuclei were stained with DAPI. Protein G polygons (green) and DAPI (blue) were monitored using confocal microscopy.

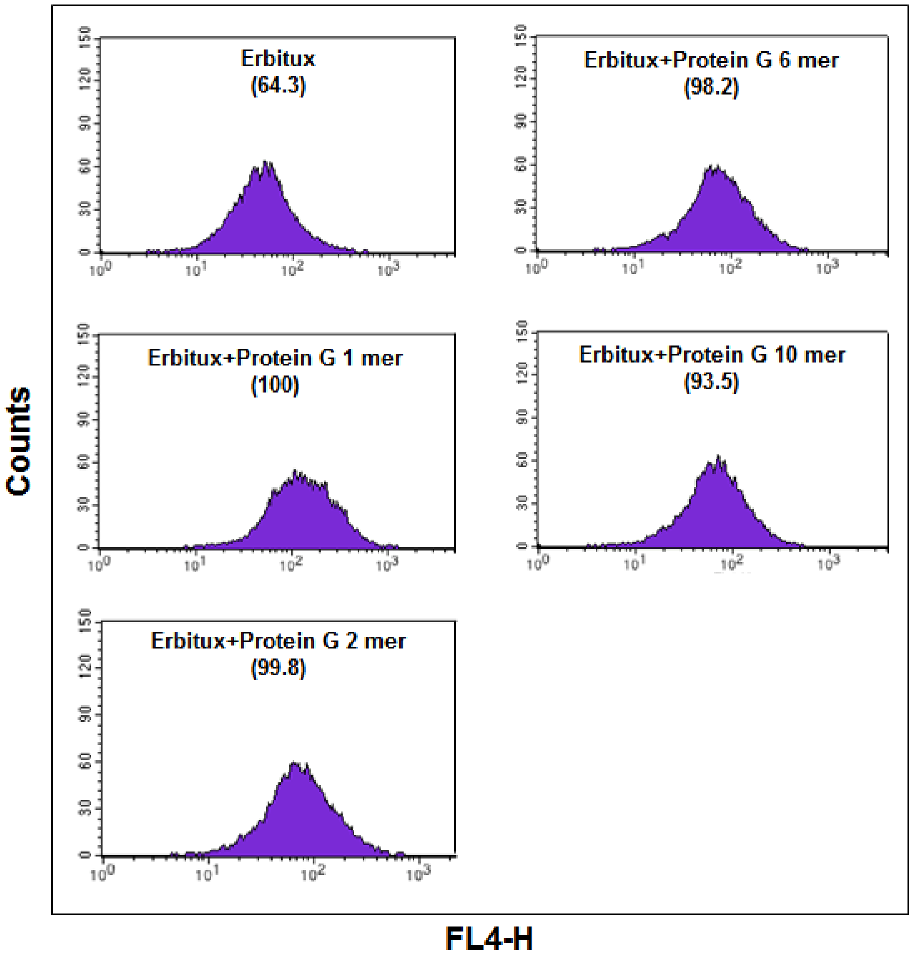

Supplementary Figure 22. Flow cytometry analysis of antibody-mediated receptor internalization after 180 min. Erbitux or Erbitux-protein G bounded cells were incubated for 180 min at 37 ℃. Internalization of Cy5-Erbitux was quantified by flow cytometry, and relative fluorescence intensities are given in the cytometry profile data.

Supplementary Table 1. Protein sequences of GFP monomer variants with different linkers

Protein	Protein sequence
mGFP L1	$(His)_6$- GFP 11- *G* -GFP 1-10
mGFP L2	$(His)_6$- GFP 11- *GG* -GFP 1-10
mGFP L3	$(His)_6$- GFP 11- *GGT*-GFP 1-10
mGFP L4	$(His)_6$- GFP 11- *GGTG* -GFP 1-10
mGFP L5	$(His)_6$- GFP 11- *GGTGGS* -GFP 1-10
mGFP L6	$(His)_6$- GFP 11- *KKK* -GFP 1-10
mGFP L7	$(His)_6$- GFP 11- *GPPPPPGG* -GFP 1-10
mGFP L8	$(His)_6$- GFP 11- *ubiqutin* -GFP *1-10*

Supplementary Table 2. Protein properties of GFP charge variants.

	Length (aa)	N_{pos}	N_{neg}	$N_{charged}$	Q_{net}
CpmGFP (wt)	252	28	31	59	-3
CpmGFP (-5)	252	28	33	61	-5
CpmGFP (-7)	252	27	34	61	-7
CpmGFP (-9)	252	26	35	61	-9
CpmGFP (-15)	252	24	39	63	-15

N_{pos}, number of positively charged amino acids (Lys and Arg)

N_{neg}, number of negatively charged amino acids (Glu and Asp)

$N_{charged}$, total number of charged amino acids

Q_{net}, theoretical net charged at neutral pH

Supplementary Note 1. Protein sequences

GFP monomer (net charge -3, wild type)

MGSSHHHHHHSSGLVPRGSHMRDHMVLHEYVNAAGITGGTKGEELFTGVVPILVELDGDVNGHKFSVRGEGEGDATIGK
LTLKFICTTGKLPVPWPTLVTTLTYGVQCFSRYPDHMKRHDFFKSAMPEGYVQERTISFKDDGKYKTRAVVKFEGDTLVNR
IELKGTDFKEDGNILGHKLEYNFNSHNVYITADKQKNGIKANFTVRHNVEDGSVQLADHYQQNTPIGDGPVLLPDNHYLSTQ
TVLSKDPNEK

Protein sequences of GFP monomer are shown with GFP 11(red), GFP 1-10 (blue) and linker (underlined).

CapGFP

MGSSHHHHHHSSGLVPRGSHMRDHMVLHEYVNAAGITGGTKGEELFTGVVPILVELDGDVNGHEFSVRGEGEGDATIGEL
TLKFICTTGELPVPWPTLVTTLTHGVQCFSRYPDHMKRHDFFKSAMPEGYVQERTISFKDDGKYKTRAVVKFEGDTLVNRIE
LKGTDFKEDGNILGHKLEYNFNSHDVYITADKQENGIKAEFTVRHNVEDGSVQLADHYQQNTPIGDGPVLLPDDHYLSTET
VLSKDPNEKRDHMVLHEYVNAAGIT

Protein sequences of GFP monomer are shown with GFP 11(red), GFP 1-11 (blue) and linker (underlined).

MCherry, protein G and MBP fused GFP monomer

Protein fusions are shown in blue. Linkers are in underlined.

MCherry-GFP monomer

MGSSHHHHHHSSGLVPRGSHMMVSKGEEDNMAIIKEFMRFKVHMEGSVNGHEFEIEGEGEGRPYEGTQTAKLKVTKGG
PLPFAWDILSPQFMYGSKAYVKHPADIPDYLKLSFPEGFKWERVMNFEDGGVVTVTQDSSLQDGEFIYKVKLRGTNFPSD
GPVMQKKTMGWEASSERMYPEDGALKGEIKQRLKLKDGGHYDAEVKTTYKAKKPVQLPGAYNVNIKLDITSHNEDYTIVE
QYERAEGRHSTGGMDELYKGSRDHMVLHEYVNAAGITGGTKGEELFTGVVPILVELDGDVNGHEFSVRGEGEGDATIGEL
TLKFICTTGELPVPWPTLVTTLTHGVQCFSRYPDHMKRHDFFKSAMPEGYVQERTISFKDDGKYKTRAVVKFEGDTLVNRIE
LKGTDFKEDGNILGHKLEYNFNSHDVYITADKQENGIKAEFTVRHNVEDGSVQLADHYQQNTPIGDGPVLLPDDHYLSTET
VLSKDPNEK

Protein G-GFP monomer

MGSSHHHHHHSSGLVPRGSHMRDHMVLHEYVNAAGITGGTKGEELFTGVVPILVELDGDVNGHEFSVRGEGEGDATIGEL
TLKFICTTGELPVPWPTLVTTLTHGVQCFSRYPDHMKRHDFFKSAMPEGYVQERTISFKDDGKYKTRAVVKFEGDTLVNRIE
LKGTDFKEDGNILGHKLEYNFNSHDVYITADKQENGIKAEFTVRHNVEDGSVQLADHYQQNTPIGDGPVLLPDDHYLSTET
VLSKDPNEKGSGGTTYKLVINGKTLKGETTTKAVDAETAEKAFKQYANDNGVDGVWTYDDATKTFTVTE

N-terminal fused MBP-GFP monomer

MGSSHHHHHHSSGLVPRGSHMMKIEEGKLVIWINGDKGYNGLAEVGKKFEKDTGIKVTVEHPDKLEEKFPQVAATGDGPDI
IFWAHDRFGGYAQSGLLAEITPDKAFQDKLYPFTWDAVRYNGKLIAYPIAVEALSLIYNKDLLPNPPKTWEEIPALDKELKAKG
KSALMFNLQEPYFTWPLIAADGGYAFKYENGKYDIKDVGVDNAGAKAGLTFLVDLIKNKHMNADTDYSIAEAAFNKGETAMT
INGPWAWSNIDTSKVNYGVTVLPTFKGQPSKPFVGVLSAGINAASPNKELAKEFLENYLLTDEGLEAVNKDKPLGAVALKSY
EEELAKDPRIAATMENAQKGEIMPNIPQMSAFWYAVRTAVINAASGRQTVDEALKDAQTNSSSNNNNNNNNNNLGIEGRGG
SRDHMVLHEYVNAAGITGGTKGEELFTGVVPILVELDGDVNGHEFSVRGEGEGDATIGELTLKFICTTGELPVPWPTLVTTLT
HGVQCFSRYPDHMKRHDFFKSAMPEGYVQERTISFKDDGKYKTRAVVKFEGDTLVNRIELKGTDFKEDGNILGHKLEYNFN
SHDVYITADKQENGIKAEFTVRHNVEDGSVQLADHYQQNTPIGDGPVLLPDDHYLSTETVLSKDPNEK

C-terminal fused MBP-GFP monomer

MGSSHHHHHHSSGLVPRGSHMRDHMVLHEYVNAAGITGGTKGEELFTGVVPILVELDGDVNGHEFSVRGEGEGDATIGEL
TLKFICTTGELPVPWPTLVTTLTHGVQCFSRYPDHMKRHDFFKSAMPEGYVQERTISFKDDGKYKTRAVVKFEGDTLVNRIE
LKGTDFKEDGNILGHKLEYNFNSHDVYITADKQENGIKAEFTVRHNVEDGSVQLADHYQQNTPIGDGPVLLPDDHYLSTET
VLSKDPNEKGSMKIEEGKLVIWINGDKGYNGLAEVGKKFEKDTGIKVTVEHPDKLEEKFPQVAATGDGPDIIFWAHDRFGGY
AQSGLLAEITPDKAFQDKLYPFTWDAVRYNGKLIAYPIAVEALSLIYNKDLLPNPPKTWEEIPALDKELKAKGKSALMFNLQEP
YFTWPLIAADGGYAFKYENGKYDIKDVGVDNAGAKAGLTFLVDLIKNKHMNADTDYSIAEAAFNKGETAMTINGPWAWSNID
TSKVNYGVTVLPTFKGQPSKPFVGVLSAGINAASPNKELAKEFLENYLLTDEGLEAVNKDKPLGAVALKSYEEELAKDPRIAA
TMENAQKGEIMPNIPQMSAFWYAVRTAVINAASGRQTVDEALKDAQTNSSSNNNNNNNNNNLGIEGR

Genetically fused protein G repeats (mono-, di- and trivalent)

Protein G (monovalent)

MGSSHHHHHHSSGLVPRGSHMTYKLVINGKTLKGETTTKAVDAETAEKAFKQYANDNGVDGVWTYDDATKTFTVTE

Protein G (divalent)

MTYKLVINGKTLKGETTTKAVDAETAEKAFKQYANDNGVDGVWTYDDATKTFTVTEKPEVIDASELTDAVTTYKLVINGKTLK
GETTTKAVDAETAEKAFKQYANDNGVDGVWTYDDATKTFTVTELEHHHHHH

Protein G (trivalent)

MGSSHHHHHHSSGLVPRGSHMMKGETTTKAVDAETAEKAFKQYANDNGVDGVWTYDDATKTFTVTEKPEVIDASELTDA
VTGSTYKLVINGKTLKGETTTKAVDAETAEKAFKQYANDNGVDGVWTYDDATKTFTVTEKPEVIDASELTDAVTTYKLVING
KTLKGETTTKAVDAETAEKAFKQYANDNGVDGVWTYDDATKTFTVTE
